# Locus coeruleus ablation in mice: protocol optimization, stereology and behavioral impact

**DOI:** 10.3389/fncel.2023.1138624

**Published:** 2023-04-27

**Authors:** Nanna Bertin Markussen, Rasmus West Knopper, Stine Hasselholt, Christian Stald Skoven, Jens Randel Nyengaard, Leif Østergaard, Brian Hansen

**Affiliations:** ^1^Center of Functionally Integrative Neuroscience (CFIN), Department of Clinical Medicine, Aarhus University, Aarhus, Denmark; ^2^Sino-Danish Center for Education and Research, University of Chinese Academy of Sciences, Beijing, China; ^3^Center for Molecular Morphology, Section for Stereology and Microscopy, Department of Clinical Medicine, Aarhus University, Aarhus, Denmark

**Keywords:** locus coeruleus, DSP-4, neurotoxin, stereology, mouse brain, behavior, noradrenaline (norepinephrine)

## Abstract

The Locus Coeruleus (LC) is in the brainstem and supplies key brain structures with noradrenaline, including the forebrain and hippocampus. The LC impacts specific behaviors such as anxiety, fear, and motivation, as well as physiological phenomena that impact brain functions in general, including sleep, blood flow regulation, and capillary permeability. Nevertheless, the short- and long-term consequences of LC dysfunction remain unclear. The LC is among the brain structures first affected in patients suffering from neurodegenerative diseases such as Parkinson’s disease and Alzheimer’s Disease, hinting that LC dysfunction may play a central role in disease development and progression. Animal models with modified or disrupted LC function are essential to further our understanding of LC function in the normal brain, the consequences of LC dysfunction, and its putative roles in disease development. For this, well-characterized animal models of LC dysfunction are needed. Here, we establish the optimal dose of selective neurotoxin N-(2-chloroethyl)-N-ethyl-bromo-benzylamine (DSP-4) for LC ablation. Using histology and stereology, we compare LC volume and neuron number in LC ablated (LCA) mice and controls to assess the efficacy of LC ablation with different numbers of DSP-4 injections. All LCA groups show a consistent decrease in LC cell count and LC volume. We then proceed to characterize the behavior of LCA mice using a light-dark box test, Barnes maze test, and non-invasive sleep-wakefulness monitoring. Behaviorally, LCA mice differ subtly from control mice, with LCA mice generally being more curious and less anxious compared to controls consistent with known LC function and projections. We note an interesting contrast in that control mice have varying LC size and neuron count but consistent behavior whereas LCA mice (as expected) have consistently sized LC but erratic behavior. Our study provides a thorough characterization of an LC ablation model, firmly consolidating it as a valid model system for the study of LC dysfunction.

## 1. Introduction

The brainstem nucleus Locus Coeruleus (LC) consists of only a few thousand neurons in mice (and about 50,000 in the human brain) (Benarroch, 2018) but influences the central nervous system via projections that release the neurotransmitter noradrenaline (NA). The LC is known to project its noradrenergic fibers to neurons, glia, and microvessels throughout the brain (Giorgi et al., 2020) and is implicated in numerous brain functions, including arousal (Ross and Van Bockstaele, 2021), the sleep-wakefulness cycle (St Louis and Boeve, 2017; Benarroch, 2018; Havekes et al., 2019), anxiety (McCall et al., 2015), attention (Aston-Jones and Cohen, 2005), and memory formation (Khakpour-Taleghani et al., 2009; Takeuchi et al., 2016), and in homeostatic processes such as trophic support (Edeline et al., 2011; Martins and Froemke, 2015) and blood flow control (Bekar et al., 2012; Toussay et al., 2013). Considering its deep involvement in many aspects of brain function, our understanding of the role of the LC in the normal brain is surprisingly lacking. Even the function of NA is being reconsidered as it is increasingly understood to overlap or function in parallel with dopamine (Ranjbar-Slamloo and Fazlali, 2020). The LC is, therefore, also considered to be implicated in neuropsychiatric conditions such as depression (Bremner et al., 1996a,b; Morris et al., 2020b) and anxiety (Morris et al., 2020a). In addition, interest in LC is growing due to the finding that LC damage may play a role in the pathogenesis of both Parkinson’s disease (Del Tredici et al., 2002) and Alzheimer’s disease (Chen et al., 2022). This is supported by the finding that LC involvement in incidental Lewy body disease precedes the formation of α-synuclein aggregates and neuronal death in midbrain substantia nigra (Del Tredici and Braak, 2013). While findings suggest that the LC may play a crucial role in the development of the diseases (Chalermpalanupap et al., 2013; Betts et al., 2019; Kelly et al., 2019), the role of LC in the etiology of these diseases is unclear.

Animal studies of LC function are critical for expanding our understanding of the intact LC-NA system and for studying the effect of LC damage on brain health. For such studies to be fruitful, there is a need for well-characterized animal models of LC dysfunction. In mice, an isolated LC ablation can be achieved by intraperitoneal injection (IP) of the neurotoxin N-(2-chloroethyl)-N-ethyl-bromo-benzylamine (DSP-4). Previous studies have estimated an optimal dose of neurotoxin to be 50 mg/kg, which damages around 90% of the LC neurons without signs of any other damage (Ross and Stenfors, 2015). However, the optimal administration protocol, according to the number of administrations, varies between studies and is not well-established. The behavioral response to DSP-4-based LC ablation and DSP-4 dose also has not been described. In our study, we set out to fill these gaps by combined investigation of the effect of DSP-4 dose on LC ablation efficacy and behavior. Three LC ablated (LCA) groups of mice were injected IP with DSP-4 either two, three, or four times at 1-week intervals. A control (CON) group received saline injections four times at the same time interval. To evaluate the effects and efficacy of the ablation, behavioral testing and, stereology were conducted. For all groups, immunohistochemically stained brain slices were used for stereological assessment of LC volume and estimation of LC neuron count. Light-dark box (LDB) and Barnes maze (BM) tests were performed to assess curiosity, anxiety-like traits, and learning ability. Time spent asleep was also monitored for CON and LCA groups. We conclude by evaluating LCA mouse behavior and stereology results against known LC functions and discuss the validity of the LCA model for future studies.

## 2. Materials and methods

### 2.1. Animals

**FIGURE 1:**
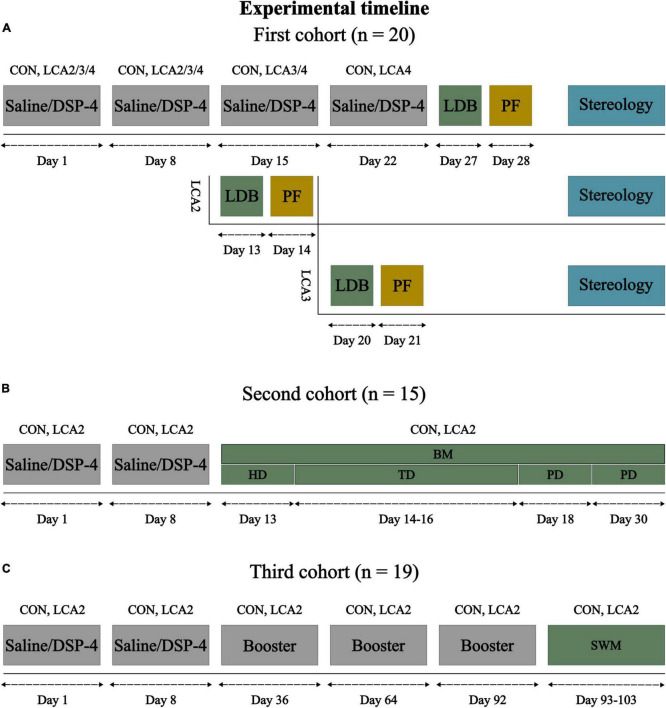
Experimental timeline for each of the three cohorts of mice. **(A)** The first cohort of mice was divided into four groups: CON (*n* = 5), LCA2 (*n* = 5), LCA3 (*n* = 5), and LCA4 (*n* = 5), receiving four saline, two DSP-4, three DSP-4, and four DSP-4 injections, respectively. All groups underwent the LDB test 5 days after the last injection. The brain tissue was perfusion fixated 1 day after the LDB test and prepared for stereology. **(B)** Based on the results of the first cohort, the second cohort was divided into a CON group (*n* = 8) and an LCA2 group (*n* = 7). These groups underwent the BM test consisting of six test days. The mice explored the BM table for 3 min during the first day (HD), in which the escape box and visual cues were removed. During the following three TDs, each mouse underwent three trials per day of 3 min or until they entered the escape box with three visual cues on the walls. The two PDs consisted of one trial of 3 min in which the escape box was removed. **(C)** The last cohort of mice was divided into a CON group (*n* = 10) and an LCA2 group (*n* = 9). The groups underwent non-invasive sleep-wakefulness monitoring (SWM) approximately 3 months after the two initial injections. Meanwhile, the CON and LCA2 groups received three booster injections of saline or DSP-4, respectively. Each SWM session lasted for 24 h. LDB, light-dark box; BM, Barnes maze; HB, habituation day; TD, training day; PD, probe day; SWM, sleep-wakefulness monitoring; PF, perfusion fixation.

All animal procedures were conducted in accordance with the ARRIVE guidelines and the European Communities Council Directive (2010/EU), and were approved by the Animal Experiments Inspectorate (Permit no: 2020-15-0201-00684) in Denmark. The data were sampled from three cohorts of mice (Figure 1). The LDB test and stereology were conducted in the first cohort of 20 male C57BL/6NTac mice (Taconic, Ejby, Denmark; 5 weeks old). At this age, indicators of the noradrenergic system have reached adult levels in rodents (Murrin et al., 2007). These mice were distributed randomly into four equally sized groups (*n* = 5 per group), one CON and three LCA groups. The BM test was performed on a second cohort of 15 male C57BL/6NTac mice (Taconic, Ejby, Denmark; 5 weeks old) divided into a CON group (*n* = 8) and an LCA group (*n* = 7). The third cohort of 19 male C57BL/6NRj mice (France; 10 weeks old) was divided into a CON group (*n* = 10) and an LCA group (*n* = 9) and underwent non-invasive sleep-wakefulness monitoring (SWM). All mice were housed by their experimental groups in individually ventilated cages under a 12-h light/dark cycle (lights on: 07:00 h) at a room temperature of 23 ± 1°C and 54 ± 2% air humidity with water and food ad libitum. The mice were handled regularly for 2 weeks before the LDB test, BM test, and SWM to ensure familiarity with the researcher.

### 2.2. Locus coeruleus ablation

The neurotoxin DSP-4 (Sigma-Aldrich, product no.: C8417) induces a long-lasting LC neurodegeneration by selectively accumulating inside the LC nerve terminals (Ross and Stenfors, 2015). The first cohort of mice was divided into four groups receiving either two (LCA2), three (LCA3), or four (LCA4) IP injections of DSP-4 (50 mg/kg) dissolved in saline (0.9% NaCl) (Figure 1A). These three LC ablated groups are collectively referred to as the LCA groups. A control (CON) group was treated with four injections of saline of similar volume instead of DSP-4. The doses were administered with 1 week of separation and freshly prepared before each injection due to the known instability of the toxin (Ross and Stenfors, 2015). Both the second and third cohorts of mice received two IP injections of either DSP-4 or saline. Additionally, the third cohort of mice received a monthly booster injection of DSP-4 or saline three times before undergoing SWM. The behavior and weight of the mice were monitored throughout the study to ensure the mice’s wellbeing and to describe potential side effects of the DSP-4.

### 2.3. Behavioral tests

To characterize and evaluate the validity of the LCA mouse model, the mice underwent an LDB test, BM test, and SWM. Before the LDB and the BM tests, the mice were placed in an acclimatization box with white background noise for 30 min. Before each session, all equipment was cleaned with a 30% alcohol solution to remove any olfactory cues. Both behavioral tests were analyzed using automated video tracking software (Noldus EthoVision XT 15). Each trial was recorded using infrared (IR) cameras (Basler, acA1300–60 gm, Ahrensburg, Germany) with IR lenses (Computar, H3Z4512CS-IR, Tokyo, Japan). The behavioral room was illuminated by two IR light sources (rayTEC, VAR2-i2-1, Ashington, United Kingdom) in addition to the visible light source.

#### 2.3.1. Light-dark box test

The LDB test consisted of a single session of 10 min aiming to assess the effect of LC ablation on the unconditional anxiety response to novel, open, and light environments (Kulesskaya and Voikar, 2014). Data were divided into time intervals of the first 5 min and the last 5 min to demask any habituation effect. The light-dark box (Noldus, Wageningen, Netherlands) is a 60 cm × 20 cm box divided into an open (light) compartment (20 cm × 40 cm) and a dark chamber (20 cm × 20 cm) separated by a 5 cm × 5 cm opening (Supplementary Figure 1A). The dark compartment is closed off with a lid that is translucent to IR light to allow video tracking of the animal also while it is inside the dark compartment. The videos were recorded at 25 fps, 5,000 μs exposure time, 1,282 × 1,026 px resolution, and analyzed with a sample rate of 12.5 Hz. The illuminance of the light compartment was approximately 120 lux. The LDB test parameters were divided into two: Anxiolytic parameters (Simon et al., 1994; Hascoët and Bourin, 1998) and motility parameters (Recober et al., 2010). Five anxiolytic parameters were included: (1) time spent in the light compartment, (2) the percentage of time in the light compartment along the walls (thigmotaxis), (3) the number of transitions from light to dark compartment, (4) latency to the enter dark compartment, and (5) latency to enter light compartment after entering the dark compartment. Three motility parameters were calculated to assess whether a difference among the anxiolytic parameters could be attributed to motor-related changes: (1) The distance moved in a compartment divided by the time spent in that compartment (mean velocity), (2) the distance moved in a compartment divided by the time spent moving in that compartment (mean velocity of movement), and (3) the time spent moving in a compartment divided by the time spent in that compartment (percentage of time moving). To avoid subtle changes in the position of the animal’s detected center point adding to distance-related parameters, the Lowess smoothing method using 10 samples before and after each sample point was applied on all tracks. The moving/not moving parameter was averaged over three samples and based on the velocity parameter, also averaged over three samples. The animal was categorized as moving once the velocity was above 2 cm/s and not moving once under 1.75 cm/s. If the detected velocity was between the thresholds, the animal was categorized as the last reached threshold. The buffer was applied to minimize the number of transitions between the categories. To avoid false transitions between compartments, a transition to a new compartment was counted once the animal was more than 3 cm from the border between the compartments. Initially, the animal was placed facing toward the back wall in the light compartment furthest away from the dark compartment. The tracking was initiated once the automated tracking system had a stable detection of the animal and the experimenter was out of the defined area.

#### 2.3.2. Barnes maze test

The BM test was used to study the effect of LCA on spatial learning and memory (Gawel et al., 2019) in the second cohort of mice. The BM table (Ugo Basile, Gemonio, Italy) consists of a circular table of 1 m in diameter with 20 holes evenly distributed along the periphery (Supplementary Figure 1B). Each hole is 5 cm in diameter. Entry zones were defined as an 8 cm in diameter circle around the holes, except from the escape box, where the entry zone diameter was set to 12 cm (Supplementary Figure 1C). Camera settings were similar to the LDB test except for the exposure time of 40,000 μs per frame. The experimental protocol was divided into three: (1) one habituation day (HD) with 3 min of exploration of the Barnes maze table without the escape box and visual cues, (2) three training days (TDs) of a maximum of 3 min or until entering the escape box, and (3) two probe days (PDs) of 3 min duration without the escape box. The PDs were separated by 1 week to evaluate the retention of acquired information (Figure 1B). Escape box was kept at the same position during all TDs. Initially, the animals were placed in a cylindrical box which was gently placed upturned in the center of the BM table on all test days. Tracking of the mouse center point was initiated once the cylindrical box was removed, the mouse’s center point was steadily detected, and the experimenter was out of the defined arena. The three visual cues were mounted on the walls around the table for the mice to navigate during the TDs and PDs. To assess spatial memory, four parameters were chosen: (1) Time before escape, (2) time in entry zone relative to total time spent, (3) number of visits to non-target zones, and (4) latency to entry zone. To evaluate differences in search strategies between the groups, number of different non-target visits and time in each quadrant relative to total time spent was also analyzed. The BM test tracks were smoothened similar to the LDB test, and velocity and movement parameters were averaged over five samples but otherwise similar to the LDB test. All entry zones had an exit threshold of 4 cm. For both the LDB and DM tests, the pixel color of the heatmaps represents the average proportion of each group’s tracks found at that pixel. The color bar was scaled to the maximum pixel value across all groups and the two analyzed intervals.

#### 2.3.3. Sleep-wakefulness monitoring

The non-invasive SWM was conducted using a PiezoSleep Mouse Behavioral Tracking System (Signal Solutions, Lexington, KY, USA) with a piezoelectric film covering the cage floor (Supplementary Figure 2D). During SWM, the mechanical pressure on the piezoelectric film is recorded by PiezoSleep (Signal Solutions, Lexington, KY, USA; software v. 2.18) and subsequently analyzed using SleepStats (Signal Solutions, Lexington, KY, USA; software v. 2.18). An algorithm classified the animal as being asleep based on the pressure signal patterns from the thorax during respiration. The sleep cages were positioned on a concrete surface to minimize external vibrations which might contaminate the recordings. All monitoring was done in an animal stable with minimal disturbances and equivalent environmental conditions to the animals’ daily stable. The mice were placed in the sleep cage for 15 min before starting SWM which lasted for 24 h. All SWM sessions were initiated between approximately 12:00 and 15:00 h.

### 2.4. Sample preparation and sectioning

The day after the LDB test, anesthesia was induced with 5% isoflurane mixed with a flow of 0.2 L/min oxygen and 0.2 L/min air, followed by an IP injection of 0.5 mL pentobarbital (400 mg/ml, Alfasan, Woerden, Netherlands). After a thoracotomy, the mice were perfused with heparinized saline (1:50 dilution of 5,000 IE/ml heparin, Panpharma 482,480) until the liver appeared pale (typically 2–3 min), and 4% paraformaldehyde in buffered aqueous solution (CAS. 50-00-0, VWR International, LLC.) until muscle twitching ceased. The brain was excised and post-fixated in 4% paraformaldehyde (as used above for perfusion) for 24 h, followed by dehydration in a 30% sucrose solution (CAS. 57-50-1, Sigma Aldrich, St. Louis, MO, USA) for 48 h. The brains were divided in two by a coronal cut 3 mm caudal to bregma, rapidly frozen by immersion in 2-methylbutane (Isopenthane, CAS. 78-78-4, Sigma-Aldrich, St. Louis, MO, USA) for up to 20 s, and stored at −80°C. Two hours prior to sectioning, the brain tissue was moved to the cryostat (CryoStar NX70, Thermo Fisher, Runcorn, UK) for acclimatization to −20°C. Immediately before sectioning, the caudal part of the brain was mounted on a metal plate at −12°C with the frontal cut surface down using MilliQ. Series of 50 μm sections of LC were sampled systematic uniform random with a section sampling fraction (SSF) of 1/5. A random series was Nissl stained (described below), and the remaining were series stored in cryoprotectant at −20°C.

### 2.5. Immunohistochemistry

Free floating sections were washed 3 × 5 min in Tris-Buffered Saline (TBS) with Triton (TBS-T) (0.05 M TBS, pH 7.4 + 0.3% Triton X-100), and 30 min heat induced epitope retrieval (Agilent Dako S1699) performed at 85°C in water bath, followed by 30 min cooldown. After 2 × 5 min in TBS-T and 1 × 5 min in TBS, endogenous peroxidase was quenched with 3% H_2_O_2_ in TBS, pH 7.4 for 10 min, followed by washing 10 min in TBS and 2 × 10 min in TBS-T. Sections were blocked for 30 min with normal goat serum (10%) in TBS-T and stained with primary anti-tyrosine hydroxylase (1:10.000, ab112, rabbit polyclonal, Abcam) over-night at 4°C on a shaker. After 3 × 10 min wash in TBS-T, sections were incubated with the secondary antibody (anti-rabbit, 1:400, HRP-labeled) for 2 h at room temperature on a shaker. The sections were washed 3 × 10 min in TBS-T and developed with 3,3′-Diaminobenzidine (DAB, CAS. 868272-85-9, Sigma-Aldrich, St Louis, MO, USA). To ensure tissue penetration, DAB treatment was in two steps, first for 7 min without hydrogen peroxide [DAB mixture 1: 31.5 μL DAB stock (2.5%) mixed with 8.47 mL TBS] and then for 10 min with the catalyzer (0.2 μL 30% H_2_O_2_ added per mL of DAB mixture 1). Development was stopped by rinsing the sections in TBS. Then, the sections were mounted on Superfrost glass slides (Fischer Thermo Scientific, Waltham, MA, USA) using 0.5% gelatin with 0.05% chrome alum. Sections were dried at room temperature for 20 min, rehydrated for 15 min in distilled water, and dehydrated in a series of ethanol solutions (1 min in 70%; 2 min in 96%; 2 × 5 min in 99%). After clearing for 5 + 10 min in xylene, coverslips (24 × 50 mm, #0, Menzel-Gläser, Hounisen) were mounted with Eukitt^®^ Quick-hardening mounting medium (CAS. 25608-33-7, Sigma-Aldrich, Steinheim, DE).

### 2.6. Histochemistry

For anatomical overview, one series per brain was Nissl stained. After cryo-sectioning, the sections were mounted directly on SuperFrost Plus glass adhesion slides (Epredia), dried for 30 min, and kept at −20°C until staining. Sections were stained with 0.125% Thionin (Sigma-Aldrich, cat. no. 861340, USA) for 60 s, dehydrated in a graded series of ethanol solutions (1 min in 70%; 2 min in 96%; 2 × 5 min in 99%), and cleared for 5 + 10 min in xylene. Coverslips (24 × 50 mm, #0, Menzel-Gläser, Hounisen) were mounted with Eukitt^®^ Quick-hardening mounting medium (CAS. 25608-33-7, Sigma-Aldrich, Steinheim, DE).

### 2.7. Stereology

All analyses were performed in newCAST (Visiopharm, Hørsholm, DK) using an Olympus microscope (BX51TF, Olympus Denmark, Ballerup, DK) equipped with a Prior motorized x–y specimen stage (H101BX, Prior Scientific Instruments Ltd, Fulbourn, UK), and an Olympus DP70 digital color camera (Olympus Denmark, Ballerup, DK). A z-axis analysis (Supplementary Figure 2) was conducted to ensure complete penetration of anti-tyrosine hydroxylase through the whole section thickness (Andersen and Gundersen, 1999). The brain tissue from one mouse from each LCA2 and LCA4 was excluded from the stereological analysis due to incomplete section series. Both volume and neuron count estimation were carried out blinded.

#### 2.7.1. LC volume estimation

The total LC volume was estimated using the Cavalieri estimator. The analysis was performed using a 10x objective (NA 0.40, UPlanApo, Olympus Denmark, Ballerup, DK). The two-dimensional nucleator was used for estimating the area of LC on individual sections (Gundersen, 1988). The area of interest was outlined, the center of the area marked, and five randomly directed straight lines propagating from the center point with a constant angle (360/5) between them were generated by newCAST (Supplementary Figure 1E). The cross-section of the propagating lines with the outline of the area of interest was marked (black crosses), and the corresponding area was estimated. The summated area from all sections (Σ A_i_) was then multiplied by the distance between evaluated section surfaces, T, to calculate the volume, V (Kristiansen and Nyengaard, 2012):


$$\text{V}=\text{T}\times \Sigma \,{\text{A}}_{\text{i}}$$

Please note that the estimated V is the combined volume of the right and left LC.

#### 2.7.2. LC neuron count estimation

To estimate the total number of neurons in the LC, an optical fractionator design was used (West et al., 1991). Fields of view were sampled systematic uniform random within LC, and cells were sampled number-weighted with the optical disector (Gundersen, 1986). The analysis was performed using a 60× oil objective (NA 1.35, UPlanSApo, Olympus) with 50 μm between sections, x- and y-steps of 60μm, disector height of 8 μm, and a frame area of 31.62 μm × 31.62 μm (Supplementary Figure 1E). Only cells with clear tyrosine hydroxylase positive cytosol staining (TH+) and a visible soma were counted. The cell nucleus was used as the counting unit. To estimate the total number of neurons, the following equation was used (Dorph-Petersen et al., 2001; Chen et al., 2020):


$$\text{N}=\frac{1}{\text{SSF}}\times \frac{1}{\text{ASF}}\times \frac{1}{\text{HSF}}\times \Sigma \,{\text{Q}}^{-}$$

$$=\frac{1}{\text{SSF}}\times \frac{\text{x-step}\times \text{y-step}}{\mathrm{frame}\,\mathrm{area}}\times \frac{{\text{t}}_{{\text{Q}}^{-}}}{{\text{h}}_{\text{dis}}}\times \Sigma \,{\text{Q}}^{-}$$

Where ASF and HSF js area and height sampling fraction, respectively, h_dis_ is the height of the disector, and Q^–^ was the total number of cells sampled by the optical disector. Due to local variance in the final height of the sections, and as a result, the HSF, the Q^–^-weighted mean thickness of the sections, ${\text{t}}_{Q^{-}}$, was used to calculate the HSF:


tQ-=∑i(ti×qi-)∑i(qi-)

Here, t_i_ is the local height of the section and q_i_^–^ is the number of TH+ neurons counted in the i’th counting frame.

### 2.8. Statistical analysis

**FIGURE 2:**
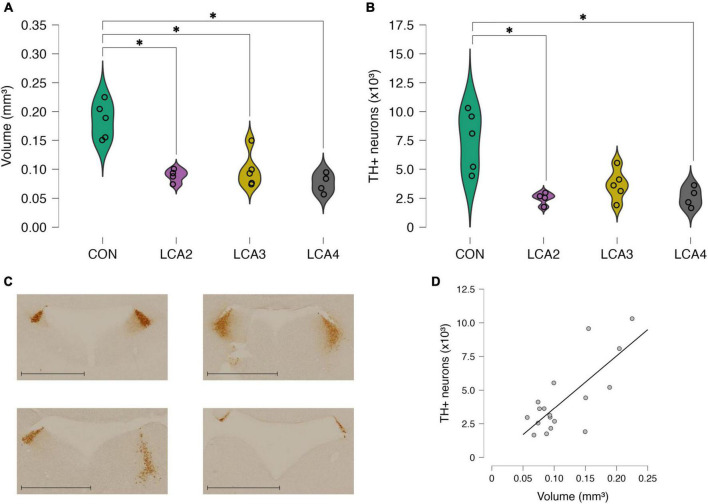
Stereological results presented as the sum of both hemispheres. **(A)** The estimated LC volume of the individual observations and smoothed distribution curves of each group. A statistically significant reduced LC volume was observed in all LCA groups compared to the CON group. No statistically significant differences were observed between the LCA groups. Group means: CON 0.185 mm^3^ [95% CI (0.157, 0.213), CV = 0.17], LCA2 = 0.089 mm^3^ [95% CI (0.078, 0.100), CV = 0.13], LCA3 = 0.099 mm^3^ [95% CI (0.072, 0.125), CV = 0.31], LCA4 = 0.076 mm^3^ [95% CI (0.059, 0.092), CV = 0.22]. **(B)** The estimated TH+ neuron count in LC of the individual observation and smoothed distribution curve of each group. A statistically significant reduction in TH+ neuron count was observed in the LCA2 and LCA3 groups compared to the CON group, while no statistically significant differences were found when comparing CON and LCA3 and the LCA groups internally. Group means: CON = 7,522 neurons [95% CI (5238, 9807), CV = 0.62, CE = 0.13], LCA2 = 2,490 neurons [95% CI (1975, 3004), CV = 0.49, CE = 0.13], LCA3 = 3664 neurons [95% CI (2495, 4833), CV = 0.53, CE = 0.16], LCA4 = 2,601 neurons [95% CI (1749, 3453), CV = 0.52, CE = 0.16]. **(C)** Representative sections of the specificity of LC with TH immunostaining. The blank area is the fourth ventricle. Upper left = CON, upper right = LCA2, lower left = LCA3, lower right = LCA4. Scale bar = 1,000 μm. **(D)** Correlation between LC volume and TH+ LC neuron count. A statistically significant correlation was found between the two parameters. CON = four saline injections (*n* = 5), LCA2 = two DSP-4 injections (*n* = 4), LCA3 = three DSP-4 injections (*n* = 5), LCA4 = four DSP-4 injections (*n* = 4). *p < 0.05.

After sampling the data, we observed an apparent systematic heterogeneity in the variance between the CON and the LCA groups (Figures 2A, B). During the initial data inspection, Levene’s test for equality of variance revealed a statistically significant difference in group variance for some of the stereological and behavioral parameters. This led our focus toward the variability of the data in general. The variance was subsequently assumed to be heterogeneous for all parameters in the stereology and the LDB test. Unlike the classical one-way analysis of variance (ANOVA), Welch’s ANOVA has no assumptions concerning equality of variance and was consequently integrated into the statistical analysis procedure. The Games-Howell *post-hoc* test shares this feature and was thus used for multiple comparisons while controlling the experiment-wise error rate. As the stereology and the LDB test were done in a different experimental design than the BM test and SWM (Figure 1), a two-way mixed ANOVA was employed for those two experiments. The Holm-Bonferroni *post-hoc* test was used for multiple comparisons of statistically significant main effects on repeated measures averaged across group levels. The same *post-hoc* test was also used for pairwise comparisons of groups in case of a statistically significant interaction. Pairwise differences were only reported if observed among groups on the same test day as statistically significant main effects on the repeated measures were considered sufficient to reflect learning curves. The confidence interval (CI) of the mean difference between TDs was corrected using the Bonferroni method. For all parameters, the group mean with 95% CI was reported. The volume estimates were additionally presented with the coefficient of variation (CV), while neuron count estimates were provided with CV and coefficient of error (CE). Both stereological parameters represent the sum of both hemispheres. For all Welch ANOVAs, the F-statistic, between and within groups degrees of freedom, *p* value, and omega squared effect size (ω^2^) are provided. For each pairwise comparison, the 95% CI of the Cohen’s *d* effect size (95% CI *d*) was reported, and if the ANOVA found a statistically significant main effect, pairwise *p* value and 95% CI of group mean difference (95% CI MD) were reported too. Statistical significance was defined as *p* value < 0.05. If the assumption of sphericity was violated in the mixed ANOVA design, the Greenhouse-Geisser correction (GGC) was applied to the test statistics. Pearson’s correlation coefficient (*r*) was computed to assess linear correlations. The statistical analysis was carried out in JASP (The JASP Team, software v. 0.17.1) and G*Power (The G*Power Team, software v. 3.1.9.6) (Faul et al., 2007, 2009).

## 3. Results

### 3.1. Stereological analysis of the effect of DSP-4 on LC

#### 3.1.1. LC volume

Overall, we observed a decrease in estimated mean LC volume in the LCA groups compared to the CON group. Notably, the number of DSP-4 injections in the LCA groups did not seem to affect the extent of the ablation in terms of volume. Welch’s ANOVA revealed a statistically significant difference in mean LC volume between at least two of the four groups [*F*(3,7.54) = 13.02, *p* = 0.002, ω^2^ = 0.74]. See Figure 2A for a graphical overview of these results. The Games-Howell *post-hoc* test found a statistically significant difference in mean LC volume between CON and LCA2 [*p* = 0.005, 95% CI MD (0.040, 0.152), *d* (0.77, 6.78)], CON and LCA3 [*p* = 0.010, 95% CI MD (0.023, 0.150), *d* (0.63, 6.16)], and CON and LCA4 [*p* = 0.002, 95% CI MD (0.052, 0.166), *d* (1.06, 7.53)]. No statistically significant difference in mean LC volume was found between LCA2 and LCA3 [*p* = 0.912, 95% CI MD (–0.063, 0.044), *d* (–2.45, 1.69)], LCA2 and LCA4 [*p* = 0.592, 95% CI MD (–0.023, 0.050), *d* (–1.67, 2.71)], and LCA3 and LCA4 [*p* = 0.529, 95% CI MD (–0.032, 0.077), *d* (–1.23, 3.02)].

#### 3.1.2. LC neuron quantity

A similar pattern was observed in the estimated mean TH+ neuron quantity in the LC (Figure 2B). Fewer TH+ neurons were observed in the LCA groups compared to the CON group, while the number of DSP-4 injections had no statistically significant effect on the TH+ neuron count. Welch’s ANOVA revealed a statistically significant difference between at least two groups [*F*(3,7.41) = 5.68, *p* = 0.025, ω^2^ = 0.60]. A Games-Howell *post-hoc* test found a statistically significant difference in group means between CON and LCA2 [*p* = 0.038, 95% CI MD (384, 9681), *d* (0.34, 5.81)] and CON and LCA4 [*p* = 0.038, 95% CI MD (353, 9490), *d* (0.31, 5.71)]. No statistically significant difference was found between CON and LCA3 [*p* = 0.092, 95% CI MD (–683, 8400), *d* (–0.01, 4.74)], LCA2 and LCA3 [*p* = 0.364, 95% CI MD (–3506, 1158), *d* (–2.82, 1.38)], LCA2 and LCA4 [*p* = 0.996, 95% CI MD (–1995, 1772), *d* (–2.24, 2.10)], and LCA3 and LCA4 [*p* = 0.517, 95% CI MD (–1397, 3523), *d* (–1.44, 2.74)]. Representative brain sections of LC with TH immunostaining from each group are displayed in Figure 2C.

The mean thickness of the sections was 14.7 ± 2.03 μm and a disector of 8 μm was applied (2 to 10 μm from the section surface). Out of 617 frames analyzed in total, 205 frames contained TH+ cells and a total of 391 cells were counted (Supplementary Figure 2).

A statistically significant linear relationship [*p* < 0.001, *r* = 0.76, 95% CI (0.45, 0.90)] between LC volume and TH+ neuron count was found (Figure 2D).

### 3.2. Behavioral impact of LC ablation

The LCA groups maintained a stable increase in weight comparable with the CON (Supplementary Figure 3). In general, the ablation procedure did not seem to affect animal wellbeing. However, transient behavioral side effects were observed 1–2 days after the injection of DSP-4, after which the mice appeared normal. These effects included narrowed eyes, reduced grooming, and in rare cases, altered locomotion potentially due to the injection in the thigh. In one case, a mouse injected with DSP-4 (second cohort) was put down 2 days after the second injection due to abnormal locomotion. Another mouse injected with DSP-4 in the third cohort died unexpectedly after the third booster injection. Motility and search strategy parameters were included in the behavioral analysis to distinguish behavioral differences caused by cognitive differences from motor-related effects of the LC ablation.

#### 3.2.1. Light-dark box: anxiolytic parameters

**FIGURE 3:**
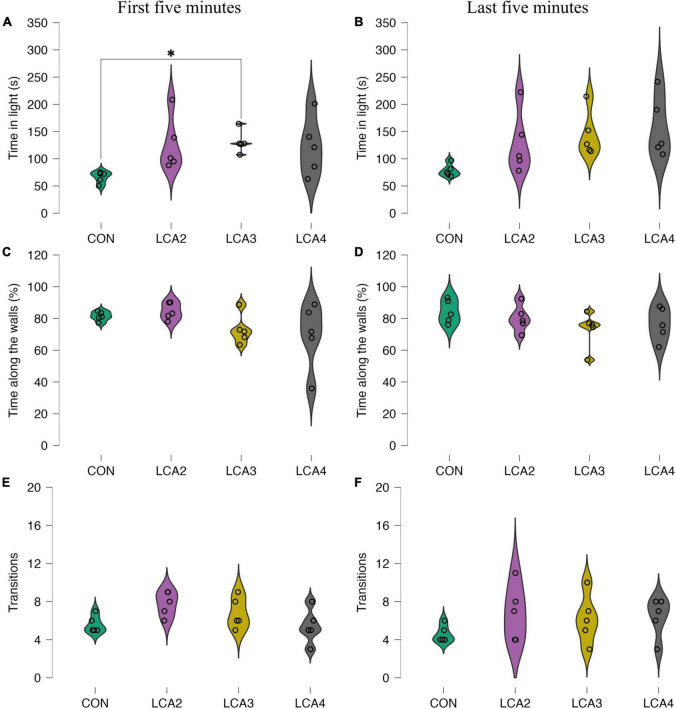
LDB test anxiolytic parameters with individual observations and smoothed distribution curves for each group segregated into the first 5 min and last 5 min. **(A,B)** The time spent in the light compartment for each group. In general, the LCA groups spent more time in the light compartment compared to the CON. A statistically significant difference was found between the CON and LCA3 during the first 5 min. **(A)** Group means: CON = 66 s [95% CI (57, 75)], LCA2 = 126 s [95% CI (83, 170)], LCA3 = 131 s [95% CI (113, 149)], LCA4 = 122 s [95% CI (75, 169)]. **(B)** The estimated group means: CON = 79 s [95% CI (67, 88)], LCA2 = 129 s [95% CI (79, 179)], LCA3 = 145 s [95% CI (108, 181)], LCA4 = 158 s [95% CI (108, 207)]. **(C,D)** The percentage of total time in the light compartment spent along the walls. **(C)** Group means: CON = 81% [95% CI (79, 84)], LCA2 = 85% [95% CI (80, 89)], LCA3 = 73% [95% CI (65, 81)], LCA4 = 70% [95% CI (51, 88)]. **(D)** Group means: CON = 84% [95% CI (78, 91)], LCA2 = 80% [95% CI (87, 73)], LCA3 = 73% [95% CI (63, 83)], and LCA4 = 77% [95% CI (67, 86)]. **(E,F)** Number of transitions from light to dark compartment. **(E)** Group means: CON = 5.6 [95% CI (4.8, 6.4)], LCA2 = 7.8 [95% CI (6.7, 8.9)], LCA3 = 6.8 [95% CI (5.4, 8.2)], and LCA4 = 5.4 [95% CI (3.8, 7.0)]. **(F)** Group means: CON = 4.6 [95% CI (3.8, 5.4)], LCA2 = 6.8 [95% CI (4.2, 9.4)], LCA3 = 6.2 [95% CI (3.9, 8.5)], LCA4 = 6.4 [95% CI (4.6, 8.2)]. CON = four saline injections (*n* = 5), LCA2 = two DSP-4 injections (*n* = 5), LCA3 = three DSP-4 injections (*n* = 5), LCA4 = four DSP-4 injections (*n* = 5). *p < 0.05.

In general, the LCA groups spent more time in the light compartment compared to the CON with no habituation effects between the first and last 5 min of the test. Our LDB results show altered but inconsistent behavior in the LCA animals compared to the CON, in which the animals exhibit only small variation (Figures 3A–F). For the first 5 min (Figure 3A), Welch’s ANOVA showed a statistically significant difference in group means [*F*(3,7.69) = 13.07, *p* = 0.002, ω^2^ = 0.24]. The Games-Howell *post-hoc* test revealed a statistically significant difference between CON and LCA3 [*p* = 0.003, 95% CI MD (–100, –28), *d* (–3.78, 0.42)]. No statistically significant difference was found between CON and LCA2 [*p* = 0.166, 95% CI MD (–149, 29), *d* (–3.64, 0.51)], CON and LCA4 [*p* = 0.234, 95% CI MD (–152, 40), *d* (–3.51, 0.60)], LCA2 and LCA3 [*p* = 0.997, 95% CI MD (–92, 83), *d* (–2.02, 1.79)], LCA2 and LCA4 [*p* = 0.999, 95% CI MD (–101, 109), *d* (–1.80, 2.01)], and LCA3 and LCA4 [*p* = 0.985, 95% CI MD (–85, 102), *d* (–1.68, 2.13)]. The analysis of the last 5 min showed similar patterns (Figure 3B). A statistically significant difference in group means was observed [*F*(3,7.25) = 6.44, *p* = 0.020, ω^2^ = 0.22]. However, the subsequent Games-Howell *post-hoc* test revealed no statistically significant pairwise differences between CON and LCA2 [*p* = 0.333, 95% CI MD (–153, 52), *d* (–3.10, 0.88)], CON and LCA3 [*p* = 0.074, 95% CI MD (–140, 8), *d* (–3.50, 0.60)], CON and LCA4 [*p* = 0.107, 95% CI MD (–180, 22), *d* (–3.85, 0.38)], LCA2 and LCA3 [*p* = 0.960, 95% CI MD (–119, 88), *d* (–2.25, 1.57)], LCA2 and LCA4 [*p* = 0.856, 95% CI MD (–144, 87), *d* (–2.56, 1.31)], and LCA3 and LCA4 [*p* = 0.974, 95% CI MD (–116, 90), *d* (–2.19, 1.62)].

To evaluate the thigmotaxis response of LC ablation, the time spent along the walls of the light compartment relative to the total time in the compartment was calculated. Though less pronounced, the tendency of more inconsistent behavior in the LCA groups compared to the CON is preserved, while a generally decreased group mean is observed in the LCA groups across time intervals. For the first 5 min (Figure 3C), Welch’s ANOVA showed no statistically significant main effect [*F*(3,7.84) = 2.08, *p* = 0.183, ω^2^ = 0.10], while the estimated effect sizes of the mean difference between CON and LCA2 was 95% CI *d* [–2.18, 1.64], CON and LCA3 was 95% CI *d* [−1.23, 2.65], CON and LCA4 was 95% CI *d* [−0.97, 2.98], LCA2 and LCA3 was 95% CI *d* [−0.99, 2.96], LCA2 and LCA4 was 95% CI *d* [−0.75, 3.29], and LCA3 and LCA4 was 95% CI *d* [−1.62, 2.20]. Similarly, no statistically significant difference was observed during the last 5 min [*F*(3,8.77) = 1.16, *p* = 0.378, ω^2^ = 0.04] (Figure 3D). The estimated effect size of group mean differences for CON and LCA2 was 95% CI *d* [−1.47, 2.36], CON and LCA3 was 95% CI *d* [−0.84, 3.17], CON and LCA4 was 95% CI *d* [−1.15, 2.75], LCA2 and LCA3 was 95% CI *d* [−1.22, 2.66], LCA2 and LCA4 was 95% CI *d* [−1.55, 2.27], and LCA3 and LCA4 was 95% CI *d* [−2.27, 1.55].

The number of transitions between the compartments was generally higher in the LCA groups with varying behavior in both time intervals. For the first 5 min (Figure 3E), Welch’s ANOVA found no statistically significant difference between group means [*F*(3,8.55) = 3.28, *p* = 0.076, ω^2^ = 0.23]. The estimated effect size of the pairwise differences for CON and LCA2 was 95% CI *d* [−3.57, 0.56], CON and LCA3 was 95% CI *d* [−2.78, 1.13], CON and LCA4 was 95% CI *d* [−1.77, 2.04], LCA2 and LCA3 was 95% CI *d* [−1.25, 2.62], LCA2 and LCA4 was 95% CI *d* [−0.45, 3.74], and LCA3 and LCA4 was 95% CI *d* [−1.01, 2.93]. For the last 5 min (Figure 3F), no statistically significant main effect was found [*F*(3,7.87) = 1.74, *p* = 0.238, ω^2^ = 0.00]. The effect sizes of the pairwise differences for CON and LCA2 was 95% CI *d* [−2.94, 1.00], CON and LCA3 was 95% CI *d* [−2.65, 1.23], CON and LCA4 was 95% CI *d* [−2.74, 1.15], LCA2 and LCA3 was 95% CI *d* [−1.64, 2.17], LCA2 and LCA4 was 95% CI *d* [−1.73, 2.08], and LCA3 and LCA4 was 95% CI *d* [−1.99, 1.82].

**FIGURE 4:**
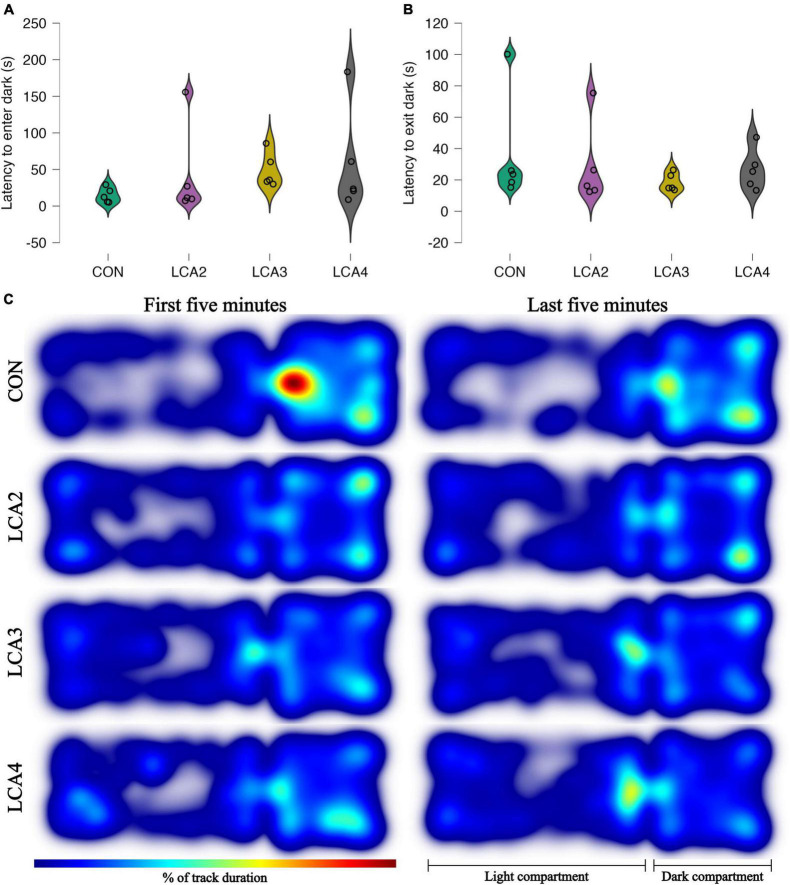
**(A,B)** LDB test anxiolytic parameters with individual observations and smoothed distribution curves for each group. **(A)** The time spent in the light compartment before the first visit to the dark compartment. Group means: CON = 15 s [95% CI (6, 24)], LCA2 = 42 s [95% CI (–14, 98)], LCA3 = 49 s [95% CI (28, 70)], LCA4 = 59 s [95% CI (–4, 123)]. **(B)** The time spent in the dark compartment before the first transition to the light compartment. CON = 37 s [95% CI (5, 68)], LCA2 = 29 s [95% CI (5, 52)], LCA3 = 19 s [95% CI (13, 24)], LCA4 = 27 s [95% CI (15, 38)]. **(C)** The heatmaps of each animal merged within groups and time intervals. The color intensity of the heatmaps is scaled relative to the maximum observed value of 8.5%. The LCA groups cover more of the light compartment, while the CON has all its peaks in the dark compartment. The overall tendency between the groups is similar in both time intervals.

The time spent in the light compartment before the first visit to the dark compartment showed no clear patterns besides more inconsistency in the LCA groups (Figure 4A). Welch’s ANOVA found no statistically significant difference between group means [*F*(3,7.47) = 3.08, *p* = 0.095, ω^2^ = 0.00]. The estimated effect sizes of group mean differences for CON and LCA2 was 95% CI *d* [−2.48, 1.37], CON and LCA3 was 95% CI *d* [−2.63, 1.25], CON and LCA4 was 95% CI *d* [−2.86, 1.06], LCA2 and LCA3 was 95% CI *d* [−2.04, 1.77], LCA2 and LCA4 was 95% CI *d* [−2.26, 1.57], and LCA3 and LCA4 was 95% CI *d* [−2.12, 1.70]. When comparing the group means of the latency to exit the dark compartment (Figure 4B), no statistically significant differences were observed [*F*(3,7.54) = 0.91, *p* = 0.482, ω^2^ = 0.00]. The effect size of the difference between CON and LCA2 was 95% CI *d* [−1.57, 2.25], CON and LCA3 was 95% CI *d* [−1.17, 2.72], CON and LCA4 was 95% CI *d* [−1.49, 2.35], LCA2 and LCA3 was 95% CI *d* [−1.48, 2.36], LCA2 and LCA4 was 95% CI *d* [−1.81, 2.00], and LCA3 and LCA4 was 95% CI *d* [−2.26, 1.57].

When mapping the mice’s activity pattern during the LDB test, the LCA groups exhibited a more dispersed coverage of the light compartment with less stationary behavior compared to the CON, who tend to move along the edges of the light compartment, and more sedentary behavior in the dark compartment (Figure 4C).

#### 3.2.2. Light-dark box: motility parameters

**FIGURE 5:**
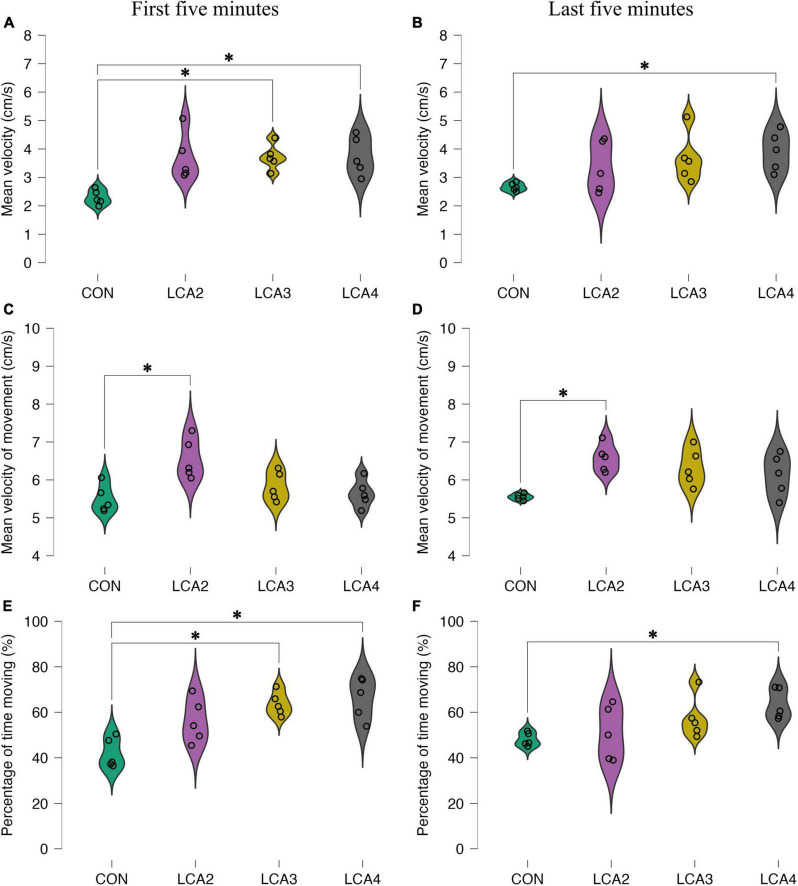
The results of the motility parameters in the dark compartment. **(A,B)** The distance moved relative to the time spent in the dark compartment. **(A)** Group means: CON = 2.3 cm/s [95% CI (2.1, 2.5)], LCA2 = 3.7 cm/s [95% CI (3.0, 4.4)], LCA3 = 3.7 cm/s [95% CI (3.3, 4.1)], LCA4 = 3.8 cm/s [95% CI (3.2, 4.4)]. **(B)** Group means: CON = 2.7 cm/s [95% CI (2.5, 2.8)], LCA2 = 3.4 cm/s [95% CI (2.6, 4.2)], LCA3 = 3.7 cm/s [95% CI (2.9, 4.4)], LCA4 = 3.9 cm/s [95% CI (3.3, 4.5)]. **(C,D)** The distance moved relative to time spent moving in a the dark compartment. **(C)** Group means: CON = 5.5 cm/s [95% CI (5.2, 5.8)], LCA2 = 6.6 cm/s [95% CI (6.1, 7.0)], LCA3 = 5.8 cm/s [95% CI (5.3, 6.0)], LCA4 = 5.6 cm/s [95% CI (5.3, 6.0)]. **(D)** Group means: CON = 5.6 cm/s [95% CI (5.5, 5.6)], LCA2 = 6.6 cm/s [95% CI (6.3, 6.9)], LCA3 = 6.3 cm/s [95% CI (5.9, 6.8)], LCA4 = 6.1 cm/s [95% CI (5.6, 6.6)]. **(E,F)** The time spent moving relative to the time spent in the dark compartment. **(E)** Group means: CON = 42% [95% CI (36, 48)], LCA2 = 56% [95% CI (48, 65)], LCA3 = 64% [95% CI (59, 68)], LCA4 = 66% [95% CI (58, 74)]. **(F)** Group means: CON = 48% [95% CI (45, 51)], LCA2 = 51% [95% CI (40, 61)], LCA3 = 58% [95% CI (49, 66)], LCA4 = 64% [95% CI (58, 70)]. *p < 0.05.

To further detail the anxiolytic parameters, three motility parameters were analyzed. Notably, while all groups appeared homogeneous in the light compartment for all motility parameters (Supplementary Figures 4A–F), substantial differences were demonstrated in the dark compartment for both time intervals (Figures 5A–F). LCA groups differ from the CON by having higher mean velocity in the dark compartment (Figures 5A, B). Furthermore, the tendency of more inconsistent behavior among the LCA groups was preserved. In the dark compartment, Welch’s ANOVA showed a statistically significant main effect during the first 5 min [*F*(3,8.12) = 15.80, *p* < 0.001, ω^2^ = 0.48]. The Games-Howell *post-hoc* test revealed a statistically significant difference between the CON and LCA3 [*p* = 0.003, 95% CI MD (−2.2, −0.6), *d* (−4.66, −0.09)], and CON and LCA4 [*p* = 0.022, 95% CI MD (−2.7, −0.3), *d* (−4.74, −0.14)]. No statistically significant difference was found between CON and LCA2 [*p* = 0.058, 95% CI MD (−2.9, 0.1), *d* (−4.63, −0.08)], LCA2 and LCA3 [*p* = 1.000, 95% CI MD (−1.5, 1.4), *d* (−1.92, 1.88)], LCA2 and LCA4 [*p* = 1.000, 95% CI MD (−1.6, 1.5), *d* (−1.99, 1.82)], and LCA3 and LCA4 [*p* = 0.999, 95% CI MD (−1.3, 1.2), *d* (−1.97, 1.84)]. A comparable pattern was observed during the last 5 min (Figure 5B). Here, a statistically significant main effect was observed [*F*(3,7.03 = 6.67, *p* = 0.018, ω^2^ = 0.22]. The *post-hoc* test found a statistically significant difference between the CON and LCA4 [*p* = 0.049, 95% CI MD (−2.5, 0.0), *d* (−3.85, 0.38)]. No statistically significant difference was found between the CON and LCA2 [*p* = 0.419, 95% CI MD (−2.3, 0.9), *d* (−2.94, 1.00)], CON and LCA3 [*p* = 0.187, 95% CI MD (−2.6, 0.6), *d* (−3.44, 0.65)], LCA2 and LCA3 [*p* = 0.945, 95% CI MD (−2.1, 1.5), *d* (−2.34, 1.49)], LCA2 and LCA4 [*p* = 0.705, 95% CI MD (−2.2, 1.1), *d* (−2.72, 1.18)], and LCA3 and LCA4 [*p* = 0.958, 95% CI MD (−1.8, 1.4), *d* (−2.25, 1.57)].

Similarly, for the mean velocity of movement, the CON and LCA groups were particularly different in the dark compartment while being more equal in the light compartment (Figures 5C, D and Supplementary Figures 4C, D). This was true for both time intervals. During the first 5 min, a statistically significant difference was found in the dark compartment [*F*(3,8.82) = 4.18, *p* = 0.042, ω^2^ = 0.45]. The *post-hoc* test showed a statistically significant difference between CON and LCA2 [*p* = 0.031, 95% CI MD (−2.0, −0.1), *d* (−4.87, −0.21)]. No statistically significant difference was observed between CON and LCA3 [*p* = 0.542, 95% CI MD (−1.1, 0.4), *d* (−2.73, 1.16)], CON and LCA4 [*p* = 0.921, 95% CI MD (−0.9, 0.6), *d* (−2.26, 1.57)], LCA2 and LCA3 [*p* = 0.144, 95% CI MD (−2.3, 0.9), *d* (−2.94, 1.00)], LCA2 and LCA4 [*p* = 0.059, 95% CI MD (0.0, 1.9), *d* (−0.04, 4.42)], and LCA3 and LCA4 [*p* = 0.864, 95% CI MD (−0.6, 0.9), *d* (−1.48, 2.36)]. Comparable differences were found during the last 5 min (Figure 5D). A statistically significant main effect was found in the dark compartment [*F*(3,7.05) = 14.85, *p* = 0.002, ω^2^ = 0.41]. The *post-hoc* test revealed a statistically significant difference between CON and LCA2 [*p* = 0.009, 95% CI MD (−1.7, −0.4), *d* (−4.79, −0.16)], while no statistically significant difference was found between CON and LCA3 [*p* = 0.077, 95% CI MD (−1.7, 0.1), *d* (−4.02, 0.28)], CON and LCA4 [*p* = 0.230, 95% CI MD (−1.6, 0.4), *d* (−3.45, 0.64)], LCA2 and LCA3 [*p* = 0.798, 95% CI MD (−0.6, 1.1), *d* (−1.33, 2.53)], LCA2 and LCA4 [*p* = 0.484, 95% CI MD (−0.5, 1.4), *d* (−0.91, 3.06)], and LCA3 and LCA4 [*p* = 0.933, 95% CI MD (−0.9, 1.3), *d* (−1.45, 2−39)].

Equivalent to the previous motility parameters, the percentage of time moving was especially different between the groups in the dark compartment during both time intervals (Figures 5E, F and Supplementary Figures 4E, F). A statistically significant main effect was observed in the dark compartment during the first 5 min [*F*(3,8.64) = 11.50, *p* = 0.002, ω^2^ = 0.56]. The Games-Howell *post-hoc* test revealed a statistically significant difference between CON and LCA3 (*p* = 0.002, 95% CI MD = [−34, −10], *d* [−5.15, −0.35]), and CON and LCA4 (*p* = 0.007, 95% CI MD [−24, −41], *d* [−5.60, −0.58]). No statistically significant difference was found between CON and LCA2 (*p* = 0.109, 95% CI MD [−31, 3], *d* [−3.93, 0.33]), LCA2 and LCA3 (*p* = 0.484, 95% CI MD [−24, 9], *d* [−2.92, 1.02]), LCA2 and LCA4 (*p* = 0.384, 95% CI MD [−29, 9], *d* [−3.31, 0.73]), and LCA3 and LCA4 (*p* = 0.938, 95% CI MD [−19, 13], *d* [−2.25, 1.573]). During the last 5 min (Figure 5F), Welch’s ANOVA found a statistically significant main effect in the dark compartment [*F*(3,7.78) = 7.06, *p* = 0.013, ω^2^ = 0.27]. A *post-hoc* test showed a statistically significant difference between CON and LCA4 (*p* = 0.016, 95% CI MD [−27, −4], *d* [−3.99, 0.29]). No statistically significant difference was found between CON and LCA2 (*p* = 0.948, 95% CI MD [−24, 18], *d* [−2.26, 1.57]), CON and LCA3 (*p* = 0.255, 95% CI MD [−26, 7], *d* [−3.12, 0.87]), LCA2 and LCA3 (*p* = 0.768, 95% CI MD [−29, 15], *d* [−2.73, 1.17]), LCA2 and LCA4 (*p* = 0.258, 95% CI MD [−34, 8], *d* [−3.57, 0.56]), and LCA3 and LCA4 (*p* = 0.655, 95% CI MD [−23, 11], *d* [−2.67, 1.22]).

#### 3.2.3. Barnes maze: memory and learning parameters

**FIGURE 6:**
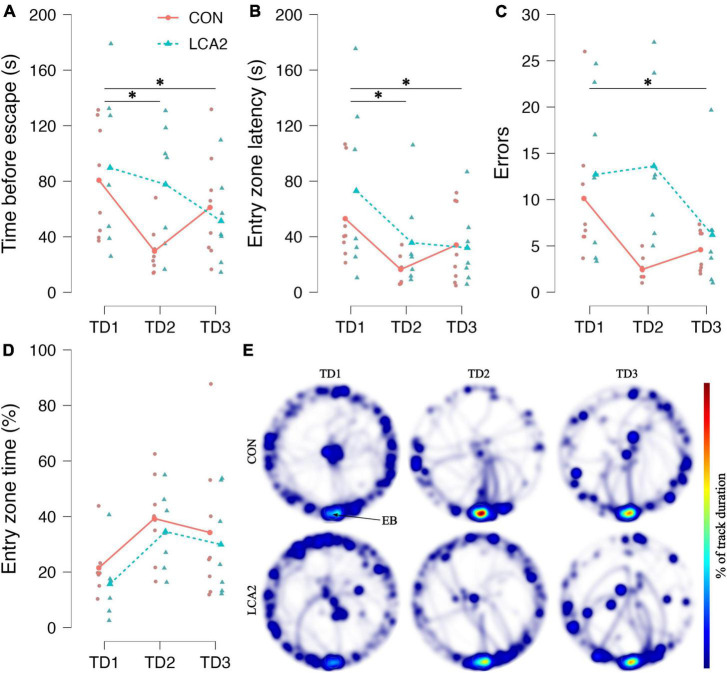
**(A)** The time before entering the escape box. Both groups showed the ability to learn across the TDs. However, the learning curve differed substantially through TD2, where LCA2 showed an increased latency to enter the escape box. TD1: CON = 81 s [95% CI (52, 108)], LCA2 = 90 s [95% CI (47, 132)], TD2: CON = 30 s [95% CI (17, 42)], LCA2 = 78 s [95% CI (45, 111)], TD3: CON = 61 s [95% CI (34, 88)], LCA2 = 51 s [95% CI (27, 76)]. **(B)** The time before the first visit to the entry zone around the escape box. Both groups reduced the latency to the first visit to the entry zone. TD1: CON = 53 s [95% CI (30, 76)], LCA2 = 73 s [95% CI (27, 119)], TD2: CON = 16 s [95% CI (9, 23)], LCA2 = 36 s [95% CI (10, 61)], TD3: CON = 34 s [95% CI (14, 54)], LCA2 = 32 s [95% CI (11, 53)]. **(C)** The number of visits to entry zones not containing the escape box. As for the latency to escape, the groups differed substantially on TD2. TD1: CON = 10.1 [95% CI (5.2, 15.1)], LCA2 = 12.7 [95% CI (6.0, 20.0)], TD2: CON = 2.5 [95% CI (1.6, 3.4)], LCA2 = 13.6 [95% CI (7.3, 20.0)], TD3: CON = 4.6 [95% CI (3.0, 6.1)], LCA2 = 6.2 [95% CI (1.5, 10.9)]. **(D)** The time spent in the entry zone relative to the total time spent. The LCA2 showed persistently less time spent in the entry zone through all TDs. TD1: CON = 20% [95% CI (12, 28)], LCA2 = 15% [95% CI (6, 25)], TD2: CON = 34% [95% CI (21, 48)], LCA2 = 28% [95% CI (16, 41)], TD3: CON = 30% [95% CI (11, 50)], LCA2 = 26% [95% CI (9, 43)]. **(E)** The average heatmaps for each group on the three TDs. The LCA2 group seems to spend less time getting into the escape box once discovered compared to the CON, which is seen to spend more time around the escape box. The color intensity of the heatmaps is scaled relative to the maximum observed value of 14%. The connected dots represent group means, while the faded, smaller dots are individual observations. EB, escape box; TD, training day. CON = two saline injections (*n* = 8), LCA2 = two DSP-4 injections (*n* = 7).

As the LCA groups were statistically indistinguishable in the stereology and the LDB test, we increased the sample size and only compared BM test performance between CON and LCA2 in a new cohort of mice. Figure 6 provides a graphical overview of these results. The mice showed the ability to learn across the TDs by reducing the latency to enter the escape box. On TD1 and TD3, the groups demonstrated comparable performances, but the learning curve through TD2 differed between the two groups, where the LCA2 spent more time before entering the escape box. A two-way mixed ANOVA found a statistically significant main effect for TDs on the latency to escape [*F*(2,26) = 4.18, *p* = 0.027, ω^2^ = 0.09]. A Holm-Bonferroni *post-hoc* test revealed a statistically significant decreased mean error from TD1 to TD2 [*p* = 0.045, 95% CI MD (1, 63), *d* (−0.07, 1.64)] and TD1 and TD3 [*p* = 0.048, 95% CI MD (−2, 60), *d* (−0.12, 1.57)]. No statistically significant difference was observed between TD2 and TD3 [*p* = 0.835, 95% CI MD (−34, 28), *d* (−0.83, 0.70)]. No statistically significant main effect between the groups was observed [*F*(1,13) = 1.06, *p* = 0.32, ω^2^ = 0.00]. Furthermore, no statistically significant interaction between the group term and TD term was found [*F*(2,26) = 2.97, *p* = 0.076, ω^2^ = 0.06].

The time before the first visit to the entry zone was also reduced over the TDs (Figure 6B). The two-way mixed ANOVA revealed a statistically significant difference between the TDs [GGC, *F*(1.37,17.49) = 6.13, *p* = 0.016, ω^2^ = 0.15]. The *post-hoc* test revealed a statistically significant difference between TD1 and TD2 [*p* = 0.008, 95% CI MD (8, 66), *d* (0.10, 1.99)] and TD1 and TD3 [*p* = 0.026, 95% CI MD (1, 59), *d* (−0.05, 1.74)]. No statistically significant difference was observed between TD2 and TD3 [*p* = 0.538, 95% CI MD (−36, 22), *d* (−1.00, 0.60)]. Neither a main effect between the groups [*F*(1,13) = 0.90, *p* = 0.361, ω^2^ = 0.00] nor an interaction [GGC, *F*(1.37,17.85) = 0.62, *p* = 0.491, ω^2^ = 0.00] were observed.

In terms of incorrect visits to entry zones not containing the escape box, a learning curve similar to the time before escape was observed (Figure 6C). The ANOVA found statistically significant differences in error count between the TDs [GGC, *F*(1.34,17.35) = 5.12, *p* = 0.028, ω^2^ = 0.12]. The difference between TD1 and TD3 was statistically significant [*p* = 0.011, 95% CI MD (1, 11), *d* (0.06, 1.84)]. No statistically significant differences were found between TD1 and TD2 [*p* = 0.171, 95% CI MD (−1, 8), *d* (−0.27, 1.33)] and TD2 and TD3 [*p* = 0.172, 95% CI MD (−2, 7), *d* (−2.18, 0.60)]. The two-way mixed ANOVA found no statistically significant main effect between groups [*F*(1,13) = 4.30, *p* = 0.059, ω^2^ = 0.11]. Similarly, the interaction was not found to be statistically significant [GGC, *F*(1.34,17.35) = 3.84, *p* = 0.056, ω^2^ = 0.08].

A small difference in the average time spent in the entry zone before entering the escape box relative to the total time spent was observed on all TDs (Figure 6D). The two-way mixed ANOVA found no statistically significant main effect on TDs [*F*(2,26) = 2.37, *p* = 0.113, ω^2^ = 0.05], groups [*F*(1,13) = 0.55, *p* = 0.472, ω^2^ = 0.00], and interaction [*F*(2,26) = 0.01, *p* = 0.989, ω^2^ = 0.00]. The average heatmaps for the two groups on each TD illustrate how the CON spent more time in the entry zone before entering the escape box (Figure 6E). Especially, the activity patterns differ between groups on TD2, where LCA2 seem to have a more dispersed coverage of the table. Notably, the LCA2 seemed to explore more and spent less time around the entrance to the escape box compared to the CON group. This dispersion was also observed in the error count (Figure 6C).

The groups underwent two PDs to explore any difference in memory retention. No statistically significant differences were observed in any of the parameters. See Supplementary Figures 5A–C for a graphical overview of these results. With the fixed track duration during the PDs, the parameters ‘distance moved’ and ‘time moving’ correspond to the motility measures in Figures 5A, B, E, F, respectively. No differences were observed among these parameters in the BM test (Supplementary Figures 5D, E). These indistinguishable outcomes during the PDs are also visible from the heatmaps (Supplementary Figure 5F).

#### 3.2.4. Barnes maze: search strategy parameters

**FIGURE 7:**
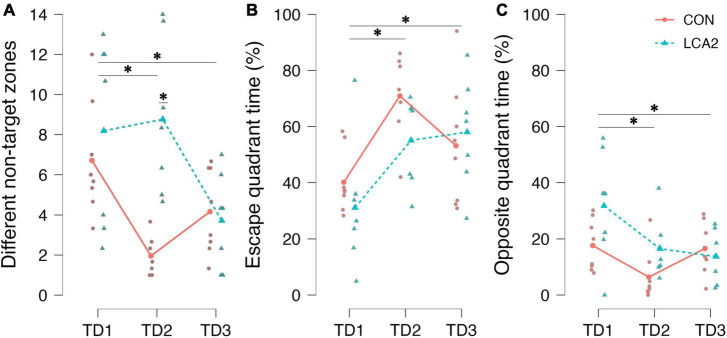
**(A)** The number of visits to different entry zones not containing the escape box. TD1 was different from TD2 and TD3. On TD2, a statistically significant difference was found between the groups. CON = 6.7 [95% CI (4.7, 8.7)], LCA2 = 8.2 [95% CI (4.7, 11.7)], TD2: CON = 2.0 [95% CI (1.3, 2.6)], LCA2 = 8.8 [95% CI (5.9, 11.6)], TD3: CON = 4.2 [95% CI (2.7, 5.6)], LCA2 = 3.7 [95% CI (2.0, 5.4)]. **(B)** The time spent in the quadrant of the arena containing the escape box relative to the total time spent. A statistically significant difference was observed between TD1 and both TD2 and TD3. TD1: CON = 40% [95% CI (32, 48)], LCA2 = 31% [95% CI (14, 48)], TD2: CON = 71% [95% CI (61, 81)], LCA2 = 55% [95% CI (43, 67)], TD3: CON = 53% [95% CI (38, 68)], LCA2 = 58% [95% CI (44, 72)]. **(C)** The time spent in the quadrant of the arena opposite to the escape quadrant relative to the total time spent in the arena. A statistically significant difference between TD1 and both TD2 and TD3 was revealed by the *post-hoc* test. A statistically significant interaction was also found in the opposite quadrant. TD1: CON = 18% [95% CI (11, 24)], LCA2 = 32% [95% CI (17, 46)], TD2: CON = 6% [95% CI (0, 13)], LCA2 = 17% [95% CI (9, 24)], TD3: CON = 17% [95% CI (10, 23)], LCA2 = 14% [95% CI (7, 21)]. *p < 0.05.

As no differences in motility parameters were observed in the light compartment of the LDB test, search strategy parameters are instead investigated in the BM test to ensure that any difference in strategy was not blurring the effect of LC ablation on the learning and memory parameters. See Figure 7 for an overview of these results. Notably, when analyzing the number of visits to entry zones not containing the escape box, the LCA2 group differs substantially from the CON on TD2 (Figure 7A). A statistically significant main effect on TDs was observed [*F*(2,26) = 10.76, *p* < 0.001, ω^2^ = 0.18]. The *post-hoc* test found a statistically significant difference between TD1 and TD2 [*p* = 0.022, 95% CI MD (−0.1, 4.0), *d* (−0.04, 1.44)] and TD1 and TD3 [*p* < 0.001, 95% CI MD (1.6, 5.5), *d* (0.29, 2.05)]. No statistically significant difference was observed between TD2 and TD3 [*p* = 0.072, 95% CI MD (−0.5, 3.4), *d* (−0.22, 1.17)]. No statistically significant main effect on groups was found [*F*(1,13) = 4.15, *p* = 0.062, ω^2^ = 0.10]. The two-way mixed ANOVA revealed a statistically significant interaction [*F*(2,26) = 12.13, *p* < 0.001, ω^2^ = 0.20]. The Holm-Bonferroni *post-hoc* test found a statistically significant difference between the groups on TD2 [*p* = 0.002, 95% CI MD (−11.8, −1.8), *d* (−4.48, −1.05)].

In our analysis, the BM table was divided into quadrants (Supplementary Figure 1C). The time spent in the quadrant containing the escape box relative to the total time spent was different on TD1 and TD2, where the LCA2 spent less time in the target quadrant (Figure 7B). Correspondingly, the LCA2 spent more time in the quadrant opposite to the escape quadrant (Figure 7C). A statistically significant main effect on TDs was observed in both the escape quadrant [*F*(2,26) = 15.21, *p* < 0.001, ω^2^ = 0.29] and opposite quadrant [*F*(2,26) = 8.59, *p* = 0.001, ω^2^ = 0.18]. Similarly, the *post-hoc* test found a statistically significant difference between TD1 and TD2 in both the escape quadrant [*p* < 0.001, 95% CI MD (−40, −14), *d* (−2.59, −0.49)] and the opposite quadrant [*p* = 0.001, 95% CI MD (5, 22), *d* (−0.23, 2.07)], and TD1 and TD3 in both the escape quadrant [*p* = 0.001, 95% CI MD (−33, −7), *d* (−2.03, −0.20)] and the opposite quadrant [*p* = 0.015, 95% CI MD (1, 18), *d* (−0.01, 1.66)] too. No statistically significant difference was found between TD2 and TD3 in both the escape quadrant [*p* = 0.161, 95% CI MD (−5, 21), *d* (−0.34, 1.17)] and the opposite quadrant [*p* = 0.273, 95% CI MD (−12, 5), *d* (−1.06, 0.42)]. The two-way ANOVA found no statistically significant difference in both the escape quadrant [*F*(1,13) = 0.88, *p* = 0.365, ω^2^ = 0.00] and the opposite quadrant [*F*(2,13) = 2.37, *p* = 0.148, ω^2^ = 0.05]. No statistically significant interaction was observed in the escape quadrant [*F*(2,26) = 2.12, *p* = 0.14, ω^2^ = 0.03], while a statistically significant interaction was found in the opposite quadrant [*F*(2,26) = 3.64, *p* = 0.040, ω^2^ = 0.07].

#### 3.2.5. Sleep-wakefulness monitoring: percentage sleep and bout duration

In general, both the CON and the LCA2 groups slept more during the light period compared with the dark period. Notably, the LCA2 group slept more during the “lights on” period compared to the CON group, while the two groups showed similar sleep percentages during the “lights off” period. See Supplementary Figure 6A for an overview. A two-way mixed ANOVA revealed a statistically significant main effect on the sleep percentage between the lights on/off intervals [*F*(1,17) = 11.19, *p* = 0.004, ω^2^ = 0.09]. No statistically significant main effect was observed between the groups [*F*(1,17) = 0.50, *p* = 0.490, ω^2^ = 0.00] nor on the interaction between the “lights on/off” intervals and groups [*F*(1,17) = 1.88, *p* = 0.188, ω^2^ = 0.01].

No clear difference in the mean duration of each sleep bout was observed between the groups (Supplementary Figure 6B). The two-way mixed ANOVA found no statistically significant main effect on time intervals [*F*(1,14) = 1.61, *p* = 0.225, ω^2^ = 0.03], groups [*F*(1,14) = 0.01, *p* = 0.332, ω^2^ = 0.00] or the interaction between time intervals and groups [*F*(1,14) = 0.13, *p* = 0.729, ω^2^ = 0.00].

## 4. Discussion

Accumulating evidence associates the LC-NA neurons with neurodegenerative diseases, including Alzheimer’s and Parkinson’s disease (Szot, 2012; Bari et al., 2020; Singh, 2020; Chen et al., 2022) and neuropsychiatric conditions (Ranjbar-Slamloo and Fazlali, 2020; David et al., 2022). It is known that NA exerts influence on cerebral blood flow regulation (Peppiatt et al., 2006; Hall et al., 2014; Giorgi et al., 2020), and recent studies suggest that microvascular dysfunction contributes to the development of psychiatric disorders (Østergaard et al., 2018) and neurodegenerative diseases (Iadecola, 2010; Kalaria, 2010; Zlokovic, 2011). Taken together, these findings may point to the LC playing a more central role in overall brain health than previously thought. Future studies will have to investigate whether the functional attributes of the LC must be reevaluated. To dissect the function of LC, effective and reliable lesion models are needed. In this study, we proposed a protocol for efficient LC ablation in mice. This mouse model can be used for investigating the isolated effect of LC dysfunction without contaminative effects from other pathologies. We have evaluated the outcome of different DSP-4 treatments by quantifying the neurodegenerative effect of either two, three, or four DSP-4 injections on LC neuron count and volume using unbiased stereology. These findings were supplemented with the behavioral results from LDB test, BM test, and SWM.

### 4.1. DSP-4 treatments induce similar reduction in LC volume and neuron count

The effect of DSP-4 was assessed using two stereological parameters: LC volume using the Cavalieri estimator and TH+ neuron count using the optical fractionator. We observed a statistically significant decrease in LC volume regardless of the number of DSP-4 injections received but no statistically significant difference between the LCA groups (Figure 2A). Similar results were found in the TH+ neuron count despite the absence of a statistically significant difference between CON and LCA3 (Figure 2B). Due to the heterogeneity of variance, Welch’s correction of the degrees of freedom was applied in the Games-Howell *post-hoc* test and thereby penalizing the test statistics. Future studies can compensate for this effect by increasing sample sizes. However, this systematic heterogeneity of variance was not expected before the data sampling, for which reason we could not take it into account in our study design. Regardless of statistical significance, the estimated effect size between CON and LCA3 is comparable with the remaining pairwise comparisons between the CON and the LCA groups. In general, all DSP-4 treatments yielded considerable estimated effect sizes on LC volume and neuron count.

Previous studies have reported DSP-4-induced LC neuron loss ranging between 20 and 60% (Fritschy and Grzanna, 1991, 1992; Zhang et al., 1995; Heneka et al., 2006). However, these studies are difficult to compare due to the methodological discrepancies, including the number of DSP-4 injections (1 vs. 2), species (mice vs. rats), sample sizes, sex, age, markers (TH vs. *D*βH), the time between treatment and tissue harvesting, and the use of secondary treatments to increase DSP-4 selectivity. We found that two, three, and four DSP-4 injections resulted in an LC neuron loss of 67, 51, and 65%, respectively, thus being at the high end of the spectrum suggested by previous studies. However, the absolute unilateral LC neuron count in these studies is lower, primarily in the control group, compared to our findings. While ranging between approximately 1,200–2,200 LC neurons in control groups and 600–1,500 LC neurons in DSP-4 treated groups (Fritschy and Grzanna, 1991, 1992; Zhang et al., 1995; Heneka et al., 2006), we found an estimated CON group mean of approximately 3800 LC neurons and between 1,200 and 1,800 LC neurons in the DSP-4 treated groups. Figure 2B shows the total count from both hemispheres. However, the previous studies did not use unbiased stereology, which could explain the incongruity in the absolute LC neuron counts. Studies utilizing unbiased stereology in LC of only wild-type mice, equaling the CON group in our study, reported 1,500–4,100 LC neurons (O’Neil et al., 2007; Liu et al., 2013; Bucci et al., 2017; Matschke et al., 2022) in agreement with our findings. Studies not using unbiased stereology in both mice and rats reported 1,500–1,800 (Goldman and Coleman, 1981; Sturrock and Rao, 1985; Loughlin et al., 1986). In general, older studies not using unbiased stereology on LC tend to underestimate LC neuron count compared to newer studies applying unbiased stereology. Similar disagreements are observed in the literature of stereological studies on the human LC, where studies show contradictory results on the effect of normal aging on LC (Lohr and Jeste, 1988; Ohm et al., 1997; Kubis et al., 2000). See review by Knopper and Hansen (2023).

Only a few of these studies also reported unbiased estimates of LC volume. While Bucci et al. (2017) estimated approximately 4,100 LC neurons with a volume of 0.035 mm^3^, Liu et al. (2019) found 1500 LC neurons with a volume of 0.100 mm^3^. Compared with our findings of 3,800 neurons with a volume of 0.093 mm^3^, it emphasizes the disagreement concerning mouse LC volume and the need for further investigation. Nevertheless, on the subject level, we found a correlation between the estimated LC neuron count and volume. The linear relationship between LC volume and neuron count was assessed using Pearson’s correlation coefficient (Figure 2D). Our results showed a statistically significant linear relationship between the two parameters. As LC is known to degenerate early in the course of both Parkinson’s disease (Bari et al., 2020; Paredes-Rodriguez et al., 2020) and Alzheimer’s disease (Singh, 2020; Chen et al., 2022), easily obtainable estimates of LC neuron count are important for early disease detection. Non-invasive estimation of LC volume in humans is possible using magnetic resonance imaging (MRI) (Sasaki et al., 2006; Clewett et al., 2016). With further refinement, such MRI methods may provide a valuable proxy for LC neuron count estimation based on the linear relationship observed in Figure 2D.

While DSP-4 administration is considered a robust and specific method for LC ablation, it should be noted that few studies have failed to show statistically significant effects of DSP-4 on LC neuron count (Szot et al., 2010), but the reliability of this result has been questioned after scrutinizing the individual NA levels (Ross and Stenfors, 2015). Yao et al. (2015) and Iannitelli et al. (2023) found effects on NA levels but no effect on LC neuron count. Here, the stereological approaches differ from ours. Our results support the use of DSP-4 for LC ablation, with consistent results seen across all LCA groups.

This study investigated the effect of different total doses of DSP-4, but assessments of single-dose sizes were beyond the scope of this paper. The single-dose dependency of NA depletion has previously been reviewed (Marien et al., 2004; Ross and Stenfors, 2015) and found 50 mg/kg IP injections of DSP-4 appear to provide sufficient effect in mice, but smaller single-doses have also been found to induce significant effects on NA levels (Heneka et al., 2002). However, readouts varied with dose. More important still, compensatory mechanisms seem to be lessened by repeated injections (Puoliväli et al., 1999), causing single injections to be of little interest for studies requiring a stable LC ablation. Perhaps for this reason, the community seems to have settled on two injections as standard, and we find it unlikely that future studies would diverge from previous studies and risk effect variability due to single injection DSP-4 administration. Instead, we wanted to assess the effect of two, three, and four injections. Importantly, we see consistent results in all three groups indicative that those studies reporting null-effect are likely affected by systematic error but also that there is no benefit of more injections than two except for the booster injections used to preserve effect as discussed below.

The incomplete removal of TH+ neurons in LC of DSP-4-treated mice raises questions about the degree of LC suppression achieved with this method. While acute effects of DSP-4 in terms of NA depletion in LC-NA projecting brain regions (Heneka et al., 2002) and enhanced inflammatory responses (Fritschy and Grzanna, 1991) have been observed within 6 h with doses as low as 50 μg/kg (Heneka et al., 2002), LC neuron loss seems to start four to 3 days after DSP-4 treatment (Fritschy and Grzanna, 1991) and progresses for at least 1 year after DSP-4 treatment (Fritschy and Grzanna, 1992). As LC neuron count seems stable in wild-type rodents across age groups until 2 years of age (Goldman and Coleman, 1981; O’Neil et al., 2007; Liu et al., 2013), the progressive LC neuron loss is presumably due to DSP-4 rather than aging. Therefore, the time between DSP-4 treatment and investigation of LC function is potentially critical. However, a compensatory mechanism is still observed with time. After the initial NA depletion in brain regions receiving LC-NA projections, NA levels gradually recover, reaching nearly pre-treatment levels in some brain regions 1 year after DSP-4 treatment (Wolfman et al., 1994; Puoliväli et al., 1999; Song et al., 2019). This is presumably due to reinnervation from surviving LC neurons (Fritschy and Grzanna, 1992). The compensatory mechanism seems reduced with the number of initial DSP-4 injections (Puoliväli et al., 1999) and has previously been obstructed using monthly booster injections of DSP-4 after the initial treatment (Heneka et al., 2010). These effects of DSP-4 seem comparable with disease models comprising LC degeneration. In wild-type mice, the inflammatory response is not affected but is exacerbated in Alzheimer’s disease models (Heneka et al., 2006; Kalinin et al., 2007) and Parkinson’s disease models (Yao et al., 2015) when using DSP-4. The loss of LC neurons in Alzheimer’s disease models (Heneka et al., 2006; O’Neil et al., 2007; Liu et al., 2013) and Parkinson’s disease models (von Coelln et al., 2004) are comparable with the effect of DSP-4. The findings of LC neuron loss in these rodent models are comparable with findings in human Alzheimer’s and Parkinson’s disease post-mortem studies (Marien et al., 2004; McMillan et al., 2011; Szot, 2012; Chen et al., 2022). In summary, this suggests that DSP-4-induced LC ablation is sufficient to exhibit comparable features with disease models, including LC neuron loss, and that further degeneration can be obtained with time concurrently with monthly injections.

The specificity of systemic administration of DSP-4 on LC-NA neurons was not investigated in this study. However, previous studies have addressed this question by measuring NA levels and *D*βH immunoreactivity in brain regions mainly innervated by LC-NA neurons and mainly innervated by non-LC-NA neurons (Fritschy and Grzanna, 1991; Heneka et al., 2006; Cassano et al., 2009; Yao et al., 2015). In general, these studies reported significant effects of DSP-4 in brain regions mainly innervated by LC-NA neurons, while the other regions were largely unaffected by DSP-4.

The TH+ neurons remaining in LC after ablation (Figure 2B) are likely dopaminergic neurons unaffected by the DSP-4, as TH is involved in the synthesis of both NA and dopamine (DA). For instance, the dopaminergic neurons in the substantia nigra and the ventral tegmental area escape degeneration after DSP-4 treatment (Heneka et al., 2006; Yao et al., 2015). Conversely, DA levels and neurons decrease in LC after ventral tegmental area lesion (Ornstein et al., 1987), suggesting that a subset of the remaining TH+ neurons are DA axon terminals originating from other dopaminergic areas.

### 4.2. LC ablation influences anxiety and explorative behavior

For further characterization of our LCA model, we used behavioral tests to supplement the stereological findings. The LDB test was used to assess anxiety-like behavior (Kulesskaya and Voikar, 2014), and the BM test for the assessment of spatial learning and memory (Gawel et al., 2019). For both time intervals analyzed, the LCA groups spent more time in the light compartment during the LDB test compared to the CON group (Figures 3A, B). Increased duration in the light compartment indicates reduced anxiety-like behavior due to the deviation from the innate aversion to brightly illuminated, open areas in mice (Kulesskaya and Voikar, 2014) and is considered a key parameter in the LDB test (Hascoët and Bourin, 1998). A statistically significant difference was observed between CON and LCA3 during the first 5 min, whereas the innate dispersion in the remaining groups impede statistically significant findings. Nevertheless, though embedded in wide CIs, the estimated effect sizes between the CON group and each LCA group emphasize the potential effects of DSP-4 on anxiety-like behavior. Surprisingly, the thigmotaxis response to LC ablation only showed a small decrease compared to the CON (Figures 3C, D). With a putative reduced anxiolytic response in accordance with the increased time spent in the light area, it was expected to see a more pronounced decrease in the relative time spent along the walls on the light compartment. In general, the LCA groups showed an increase in the number of transitions between the compartments which became more apparent during the last 5 min (Figures 3E, F). However, this difference in spontaneous explorative behavior was not statistically significant. Both latency to first transition to the dark compartment and time in the dark before entering the light compartment (Figures 4A, B) showed no consistent patterns. Heatmaps of the CON and LCA groups show dispersed activity patterns in the LCA mice in the light compartment (Figure 4C). Furthermore, the LCA groups spent less time in the corners of the dark compartment while spending more time in the opening between the two compartments compared to the CON group, indicating reduced anxiety-like behavior. The overall patterns are comparable with previous reports of reduced anxious behavior in an open field test in mice with an optogenetically inhibited LC (McCall et al., 2015). Conversely, optogenetic LC excitation induced anxious behavior. The source of this effect has been further specified to originate from the LC-prefrontal cortex projections in a study dissecting the function of different LC projections (Hirschberg et al., 2017). Future work should investigate if this finding is also an indication that the LCA mice have altered exploit/explore behavior (Addicott et al., 2017).

To ensure that motor-related changes were not causing the difference between the groups in the anxiolytic parameters, additional motility parameters were included in the analysis. Interestingly, while the groups were similar in the light compartment, all three motility parameters showed differences between the LCA groups and the CON in the dark compartment. This includes mean velocity, mean velocity of movement, and percentage of time moving (Figure 5). A potential reason for not observing this difference in the light compartment could be the relatively higher anxiolytic response in CON, masking increased ambulation among the LCA groups. However, the increased time spent in the light compartment, equal time along the walls, and increased number of transitions suggest increased explorative behavior. As seen from the heatmaps, the LCA groups spent less time being stationary in the dark compartment but keep exploring the compartment unlike the CON (Figure 4C). Previous studies have emphasized the importance of novel settings when investigating the behavioral role of LC (Takeuchi et al., 2016; Wagatsuma et al., 2018). Therefore, the LDB test analysis was divided into two time intervals to prevent habituation to mask the effects of the LC ablation. No general differences were observed between the two analyses (Figure 5 and Supplementary Figure 4).

### 4.3. LC ablation affects memory and learning in novel circumstances

The BM test results showed that both groups were able to learn over the 3 days of training. This was true for both the latency to enter the escape box (Figure 6A), latency to the first visit to the entry zone (Figure 6B), and the number errors (Figure 6C), i.e., when the mice enter an entrance area with no escape box underneath. A statistically significant difference was observed between the TDs in all three parameters. No statistically significant interaction effect was revealed for any of the parameters, but a notable effect size was observed in the interaction between treatment and TDs on latency to escape and error count. In particular, this effect can be visualized on TD2 of the BM heatmaps (Figure 6E), where the groups differ in terms of dispersed activity patterns in the LCA2 group, indicating reduced memory. During the TDs of the BM test, the mice usually spend time around the escape box before entering due to the unfamiliarity of the box. That behavior is pronounced in the LCA2 group on TD2 and TD3, indicating less anxiety-like behavior. This was quantified as the time spent in the entry zone around the escape box relative to the total time spent (Figure 6D). A small difference was observed between the groups on all TDs, which is reflected in the heatmaps. Although less pronounced than in the LDB test, the LCA2 group also exhibited larger behavioral variability in the BM test compared to the CON group (Figures 6A–D). In general, these findings suggest differentiated learning curves between the two groups. It has previously been found that reversible LC inhibition prior to training influences the acquisition of reference and working memory in a Morris water maze test, while LC inhibition at other time points exerts no influence on consolidation and retrieval (Khakpour-Taleghani et al., 2009). Furthermore, optogenetic burst stimulation of LC after memory encoding tests was also found to enhance retention 24 h after (Takeuchi et al., 2016), indicating that LC plays a role in novel contextual learning, specifically facilitated by the projection between LC and the CA3 subregion of the hippocampus (Wagatsuma et al., 2018). The difference between the CON and LCA2 from TD1 to TD2 could be interpreted as impaired acquisition, while TD2 to TD3 is an indication of consolidation and retrieval of memory. This could also explain why no difference was observed between groups during the PDs of the BM test (Supplementary Figures 5A–F). Notably, the motility measures, ‘distance moved’ and ‘time moving’ obtained under the PDs showed no differences between the PDs. This speaks against the considerations of motor-related changes accounting for the difference in motility measures of the LDB test (Figure 5). However, optogenetic stimulation of LC to induce DA release during all TDs has previously been found to increase task performance on the probe day of a BM test facilitated by the co-transmission of DA (Kempadoo et al., 2016). LC lesions in Alzheimer’s disease rat models have been shown to decrease BM test performance compared to non-lesioned LC in the same rat models (Kelly et al., 2019). This effect is presumably due to the exacerbated neurodegeneration occurring because of the permissive effect of LC lesions on neuroinflammation (Heneka et al., 2006). Studies investigating the effect of DSP-4 on reference and spatial memory in the radial arm maze test and Morris water maze test found no effects of the ablation (Sirviö et al., 1994; Heneka et al., 2006). Particularly, conflicting evidence exists on LC’s role in learning and memory, possibly due to an effect in only limited steps of learning and memory formation or an overlooked importance of arousal conditions. In general, our data shows LC ablation to influence both early phases of learning and anxiety-like behavior.

To ensure that search strategies were not affecting the BM test results, we analyzed the number of visits to different entry zones not containing the escape box (Figure 7A). Interestingly, a statistically significant difference was found between the two groups on TD2, indicating serial or random search strategies (Illouz et al., 2016) in LCA2. Additionally, the LCA mice seem to mistake the quadrant opposite the escape box. The reduced time spent in the correct quadrant by LCA2 on TD2 is largely explained by the time spent in the opposite quadrant (Figures 7B, C). This searching approach might mask larger differences in the latency to escape and the latency to the first visit to the entry zone. Furthermore, the difference in time before escape is substantially larger than the latency to the first visit to the entry zone around the escape box (Figures 6A, B). These findings support the idea of LCA mice having reduced spatial memory accompanied by increased explorative behavior as they seem to keep searching after finding the escape box.

### 4.4. LC ablation does not induce gross changes in sleep patterns

Our non-invasive SWM results did not reveal an effect of LCA on time spent asleep. This is not entirely surprising as the LC is not the sole driver of sleep-wakefulness dynamics (Hobson and Pace-Schott, 2002; Pace-Schott and Hobson, 2002) and because LC activity is already strongly suppressed during sleep (Kjaerby et al., 2022). Therefore, if the LCA mice had shown large, overall sleep disturbances, the model validity would be questionable as this would indicate a wider effect of the ablation. Age is a known determinant of mouse sleep patterns (Soltani et al., 2019). For SWM, we, therefore, decided to work with fully grown mice where the effects of brain development will not affect SWM. Furthermore, we wanted to assess the influence of long-term LC dysfunction for which reason the mice were ablated and given booster shots until fully grown to suppress compensatory mechanisms influencing NA levels. There are, however, other expected outcomes of LCA that we were not able to assess with our SWM setup. As the noradrenergic output of LC, however, is involved in the regulation of REM-NREM transitions (Pace-Schott and Hobson, 2002), the LCA model is rather expected to show perturbations in relation to proportions of transitions to/from REM sleep. However, as our non-invasive sleep assessment was unable to discern between the different stages (NREM vs. REM) of sleep, we could not investigate such effects further. A change in substrain was necessitated for sleep monitoring due to supplier shortage. The choice of the NRj substrain for SWM (both CON and LCA2) was based on genealogical proximity and phenotypical overlap with NTac used in the other parts of the study (Mekada and Yoshiki, 2021). The lack of effects seen here is most likely due to the low sensitivity of the non-invasive SWM method rather than the substrain. An intriguing new study of NA function in sleep demonstrates that NA levels appear to oscillate during sleep, causing cycles of micro-arousals but only a few awakenings (Kjaerby et al., 2022). Further, when Kjaerby et al. (2022) temporarily inhibited the LC with optogenetics, they obtained a higher propensity for REM induction. With LC “chronically inhibited” in our study, a higher proportion of REM sleep could likely be expected. However, such long-term alteration in LC activity might also have additional unanticipated effects on sleep architecture. The LCA model is ideally suited to study the long-term effects of the disruption of one of the central REM sleep modulators. It is conceivable that the effects of LCA on sleep architecture in the present study are simply masked due to the coarseness of the obtained metrics. That is, future studies should employ more sensitive methods to investigate these effects in more detail.

### 4.5. Inverse effect of LC ablation in stereological and behavioral results

All LCA mice, regardless of the number of injections, exhibited the same inconsistent behavior, while the CON group exhibited consistent behavior (Figures 3–7). Interestingly, these results were diametrically opposed to the stereology where the CON group displayed much more variation than the LCA groups (Figure 2). Our data shows that the intact LC shows substantial natural variation in size and neuron count but produces consistent behavior among animals. Conversely, the ablation reduces LC to a uniform size and neuron count between all LCA animals, but the resulting dysfunctional LC causes irregular behavior deviating noticeably from normal behavior. This effect is important as consistent LC ablations seemingly yield varying behavioral outcomes, but also because the resulting inhomogeneity of variance in the CON-LCA data causes statistical challenges that should be considered when designing future studies.

Our current understanding of the neural basis of behavior is, to a large extent, based on brain lesion studies in animals. Even with today’s extensive repertoire of methods for brain manipulation, precise brain lesion studies are believed to remain important for the assessment of theories in neuroscience and neurophysiology. We conclude our discussion by using our findings to evaluate the LCA mouse model using the three categories of animal model validity (Tadenev and Burgess, 2019). Superficially, we do not expect the LCA mice to be affected as LC dysfunction is believed to have subtle effects on brain physiology and behavior. Our observations agree with this as the LCA mice gain weight, groom, and move about in ways similar to normal controls. Our LCA model, therefore, has face validity which it would not have, were the effects of LCA readily observable. When testing behavior, we see that the LCA mice exhibit an increased level of curiosity and a decreased level of anxiety. This is expected as the LCA causes a decrease in the activating NA projections from LC to brain structures such as the amygdala and hippocampus. When assessing the LC volume and neuron count, we see a clear effect of the neurotoxin. The LCA procedure thus ensures reliable LC ablation accompanied by subtle behavioral changes in agreement with the current understanding of LC function. The LCA model, therefore, has construct validity. The model’s predictive validity is much more difficult to assess because the LCA model will at first serve as a tool to investigate the effect of LC dysfunction on brain health and physiology. Such findings may then be used to elucidate the effects of human LC dysfunction, which likely does not occur in isolation but alongside other pathological processes. Multimodal imaging approaches are likely beneficial for further characterization of subtle disease models such as the LCA model studied here (Hansen, 2020). Technologically, such studies are feasible (Mikkelsen et al., 2022) even in cases where the physiological effects of anesthesia need to be avoided (Lindhardt et al., 2022). We believe that the LCA model will shed light on important disease mechanisms which are shared among mice and humans. In such cases, the LCA model does have predictive validity, but more work is needed to establish whether this is the case.

## 5. Conclusion

Our study establishes an optimal protocol for locus coeruleus ablation in mice. Such mice can be used to characterize the role of the LC in brain health and study the effect of LC dysfunction with relevance to a multitude of diseases ranging from psychiatric disorders to neurodegenerative diseases.

## Data availability statement

The raw data supporting the conclusions of this article will be made available by the authors, without undue reservation.

## Ethics statement

This animal study was reviewed and approved by the Danish Animal Experiments Inspectorate (Permit no. 2020-15-0201-00684).

## Author contributions

BH, LØ, RK, and NM designed the study. NM and RK collected the data. JN and SH advised on the stereology. RK analyzed the data and wrote the initial draft. CS advised on the analysis, interpretation of stereology, and sleep data. RK and BH interpreted results and revised the manuscript. All authors edited the manuscript and contributed to the article and approved the submitted version.

## Supplementary material

Supplementary Figure 1 **(A)** The light-dark box and its arena settings in the automated tracking system. An example of how the center point of the animal is being tracked can be seen in the image. The time along the walls was calculated by subtracting the time spent in the center area of the light compartment from the total time spent in the light compartment. **(B)** The Barnes maze table. **(C)** The arena settings of the BM test in the automated tracking system. The table was divided into quadrants and the escape box was placed under the center lowest hole during the TDs. **(D)** The sleep cage setup. **(E)** To the left is an example of estimating LC area using the two-dimensional nucleator. The green dotted line encircles the region of interest and the cross-section between the five randomly generated lines from the center point is marked with black crosses. To the right is an example of the counting frame for LC neuron count. The red and green line excludes and includes, respectively, overlapping cells.

Supplementary Figure 2 Penetration of anti-tyrosine hydroxylase in sections. The number of cells counted in different depths of the sections is shown. Cells are binned in 2 μm intervals. The disector used for neuron count estimation was placed from 2 to 10 μm. In total, 391 cells were counted. The mean thickness of the sections was 14.7 ± 2.03 μm.

Supplementary Figure 3 The weight of the third cohort of mice on the days of the two initial injections and the three booster injections.

Supplementary Figure 4 The results of the motility parameters in the light compartment. In general, no differences were observed between the groups in any time interval for all parameters. **(A,B)** The distance moved relative to the time spent in the light compartment. **(A)** Group means: CON = 4.9 cm/s [95% CI (4.6, 5.2)], LCA2 = 4.9 cm/s [95% CI (4.4, 5.4)], LCA3 = 4.6 cm/s [95% CI (4.0, 5.2)], LCA4 = 4.1 cm/s [95% CI (3.0, 5.1)]. Welch’s ANOVA found no statistically significant difference in the light compartment during the first 5 min [*F*(3,8.28) = 0.85, *p* = 0.502, ω^2^ = 0.05]. The estimated effect size between CON and LCA2 was 95% CI *d* [−1.87, 1.94], CON and LCA3 was 95% CI *d* [−1.50, 2.33], CON and LCA4 was 95% CI *d* [−0.89, 3.01], LCA2 and LCA3 was 95% CI *d* [−1.54, 2.29], LCA2 and LCA4 was 95% CI *d* [−0.92, 3.06], and LCA3 and LCA4 was 95% CI *d* [−1.25, 2.63]. **(B)** Group means: CON = 4.0 cm/s [95% CI (3.6, 4.6)], LCA2 = 4.6 cm/s [95% CI (4.1, 5.1)], LCA3 = 4.0 cm/s [95% CI (3.2, 5.0)], LCA4 = 4.0 cm/s [95% CI (3.0, 5.0)]. No main effect was found in the light compartment [*F*(3, 8.59) = 0.93, *p* = 0.468, ω^2^ = 0.00]. The estimated effect sizes between CON and LCA2 was 95% CI *d* [−2.57, 1.30], CON and LCA3 was 95% CI *d* [−1.82, 1.99], CON and LCA4 was 95% CI *d* [−1.79, 2.02], LCA2 and LCA3 was 95% CI *d* [−1.22, 2.66], LCA2 and LCA4 was 95% CI *d* [−1.19, 2.70], and LCA3 and LCA4 was 95% CI *d* [−1.87, 1.93]. **(C,D)** The distance moved relative to time spent moving in a the light compartment. **(C)** Group means: CON = 7.1 cm/s [95% CI (6.6, 7.6)], LCA2 = 7.9 cm/s [95% CI (7.3, 8.4)], LCA3 = 7.3 cm/s [95% CI (6.9, 7.7)], LCA4 = 7.0 cm/s [95% CI (6.6, 7.4)]. The Welch’s ANOVA found no statistically significant main effect in the light compartment [*F*(3, 8.78) = 2.10, *p* = 0.172, ω^2^ = 0.21]. The effect sizes between the CON and LCA2 was 95% CI *d* [−3.53, 0.58], CON and LCA3 was 95% CI *d* [−2.32, 1.51], CON and LCA4 was 95% CI *d* [−1.70, 2.12], LCA2 and LCA3 was 95% CI *d* [−0.91, 3.06], LCA2 and LCA4 was 95% CI *d* [−0.42, 3.79], and LCA3 and LCA4 was 95% CI *d* [−1.32, 2.54]. **(D)** Group means: CON = 7.1 cm/s [95% CI (6.5, 7.7)], LCA2 = 7.8 cm/s [95% CI (7.3, 8.2)], LCA3 = 7.5 cm/s [95% CI (6.9, 8.1)], LCA4 = 7.0 cm/s [95% CI (6.8, 7.2)]. The ANOVA found no statistically significant differences between any of the groups [*F*(3, 7.76) = 3.05, *p* = 0.094, ω^2^ = 0.15]. The effect size of the pairwise comparisons for CON and LCA2 was 95% CI *d* [−3.31, 0.73], CON and LCA3 was 95% CI *d* [−2.69, 1.20], CON and LCA4 was 95% CI *d* [−1.78, 2.03], LCA2 and LCA3 was 95% CI *d* [−1.38, 2.47], LCA2 and LCA4 was 95% CI *d* [−0.63, 3.46], and LCA3 and LCA4 was 95% CI *d* [−1.09, 2.83]. (E,F) The time spent moving relative to the time spent in the light compartment. **(E)** Group means: CON = 69% [95% CI (64, 74)], LCA2 = 62% [95% CI (56, 67)], LCA3 = 53% [95% CI (44, 63)], LCA4 = 57% [95% CI (43, 70)]. The Welch’s ANOVA found no statistically significant difference between the groups in the light compartment [*F*(3, 8.64) = 1.62, *p* = 0.255, ω^2^ = 0.00]. The estimated effect sizes between CON and LCA2 was 95% CI *d* [−1.22, 2.66], CON and LCA3 was 95% CI *d* [−1.28, 2.59], CON and LCA4 was 95% CI *d* [−0.92, 3.05], LCA2 and LCA3 was 95% CI *d* [−1.97, 1.84], LCA2 and LCA4 was 95% CI *d* [−1.57, 2.25], and LCA3 and LCA4 was 95% CI *d* [−1.51, 2.32]. **(F)** Group means: CON = 58% [95% CI (55, 61)], LCA2 = 59% [95% CI (55, 63)], LCA3 = 53% [95% CI (44, 63)], LCA4 = 57% [95% CI (43, 70)]. No statistically significant difference was found in the light compartment [*F*(3,8.18) = 0.41, *p* = 0.750, ω^2^ = 0.00]. The estimated effect sizes between CON and LCA2 was 95% CI *d* [−2.06, 1.75], CON and LCA3 was 95% CI *d* [−1.48, 2.36], CON and LCA4 was 95% CI *d* [−1.79, 2.01], LCA2 and LCA3 was 95% CI *d* [−1.33, 2.53], LCA2 and LCA4 was 95% CI *d* [−1.64, 2.18], and LCA3 and LCA4 was 95% CI *d* [**−**2.24, 1.58].

Supplementary Figure 5 The results from the PDs of the BM test. **(A)** Number of errors. **(B)** Number of revisits to the target zone. The escape box is removed during the PDs. Therefore, persistent visits to the zone traditionally containing the escape box reflect spatial memory. No difference was observed. **(C)** Time in the entry zone. **(D)** Distance moved. **(E)** Time moving. **(F)** Heatmaps of both groups on the two PDs. Note that the duration is fixed during the PDs as no escape box is present. Therefore, distance moved and time moving corresponds to the motility measures during TDs.

Supplementary Figure 6 The results from the SWM. **(A)** The group mean percentage time asleep during the “light on” and “lights off” periods for both the CON and LCA2 groups. A statistically significant difference was observed between time intervals. Group means: Lights on: CON = 38% [95% CI (32, 44)], LCA2 = 45% [95% CI (37, 53)], lights off: CON = 33% [95% CI (23, 43)], LCA2 = 33% [95% CI (21, 46)]. **(B)** The group mean duration of each sleep bout during “lights on” and “lights off” periods. Group means: Lights on: CON = 305 s [95% CI (262, 350)], LCA2 = 329 s [95% CI (245, 413)], lights off: CON = 297 s [95% CI (227, 368)], LCA2 = 259 s [95% CI (209, 308)]. The mean sleep bout duration during the 24 h of SWM was 300 s [95% CI (264, 335)] and 291 s [95% CI (249, 334)] for CON and LCA2, respectively. White bar = lights on. Black bar = lights off. Error bars = 95% CI. **p* < 0.05.
