# Supplementary figures and images for: Locus coeruleus ablation in mice: protocol optimization, stereology and behavioral impact
Source: Front Cell Neurosci. 2023 Apr 27;17:1138624. doi: 10.3389/fncel.2023.1138624 (PMC10172584; doi:10.3389/fncel.2023.1138624)

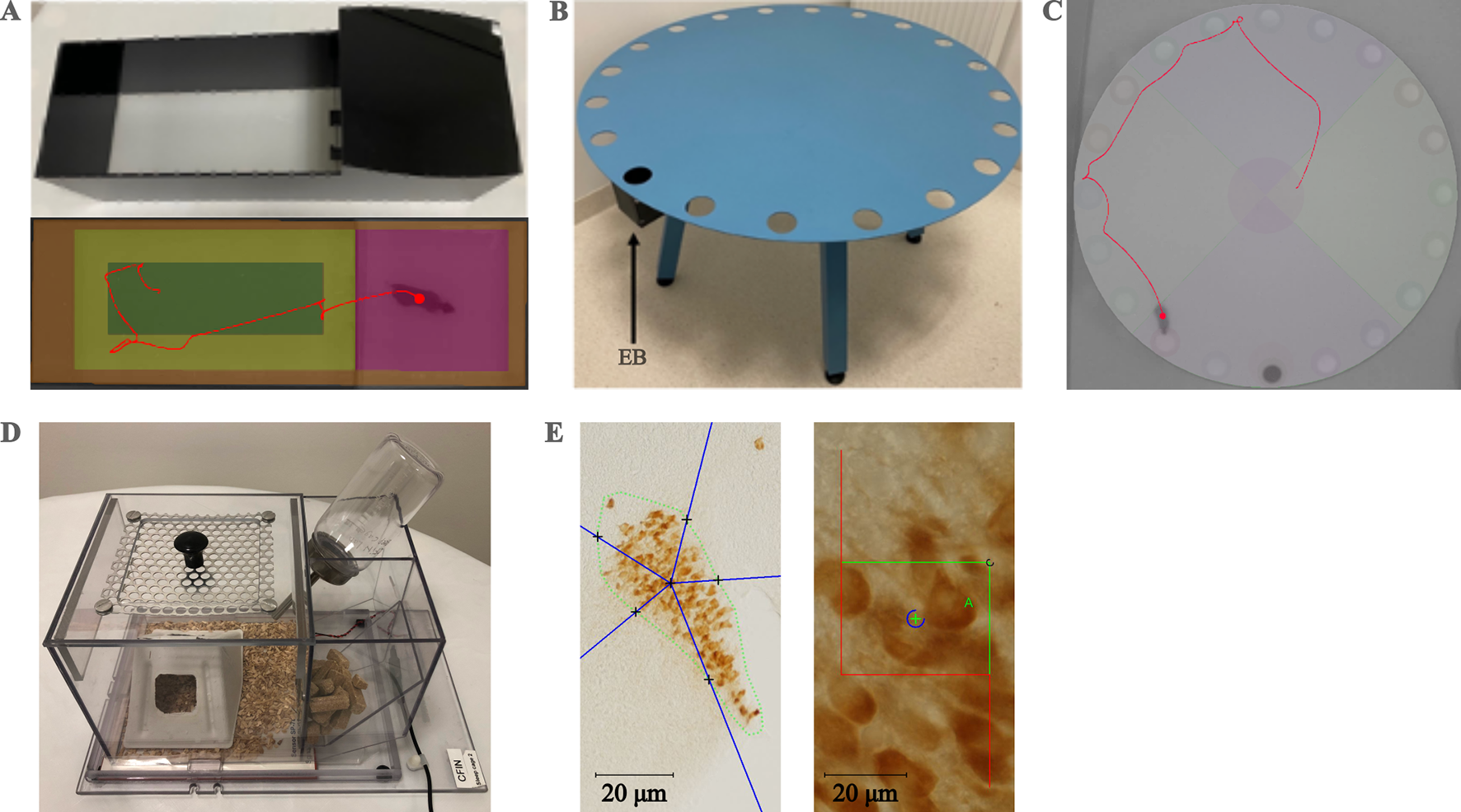

Supplement: Supplementary Figure 1 — (A) The light-dark box and its arena settings in the automated tracking system. An example of how the center point of the animal is being tracked can be seen in the image. The time along the walls was calculated by subtracting the time spent in the center area of the light compartment from the total time spent in the light compartment. (B) The Barnes maze table. (C) The arena settings of the BM test in the automated tracking system. The table was divided into quadrants and the escape box was placed under the center lowest hole during the TDs. (D) The sleep cage setup. (E) To the left is an example of estimating LC area using the two-dimensional nucleator. The green dotted line encircles the region of interest and the cross-section between the five randomly generated lines from the center point is marked with black crosses. To the right is an example of the counting frame for LC neuron count. The red and green line excludes and includes, respectively, overlapping cells. [file Image_1.TIFF]

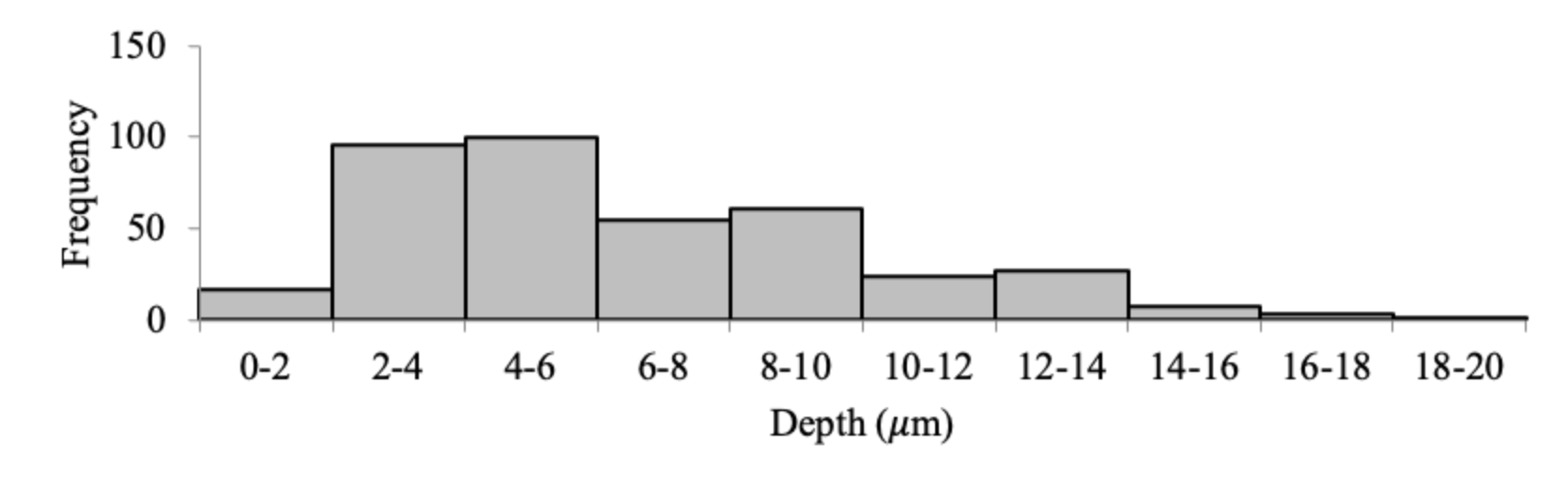

Supplement: Supplementary Figure 2 — Penetration of anti-tyrosine hydroxylase in sections. The number of cells counted in different depths of the sections is shown. Cells are binned in 2 μm intervals. The disector used for neuron count estimation was placed from 2 to 10 μm. In total, 391 cells were counted. The mean thickness of the sections was 14.7 ± 2.03 μm. [file Image_2.TIFF]

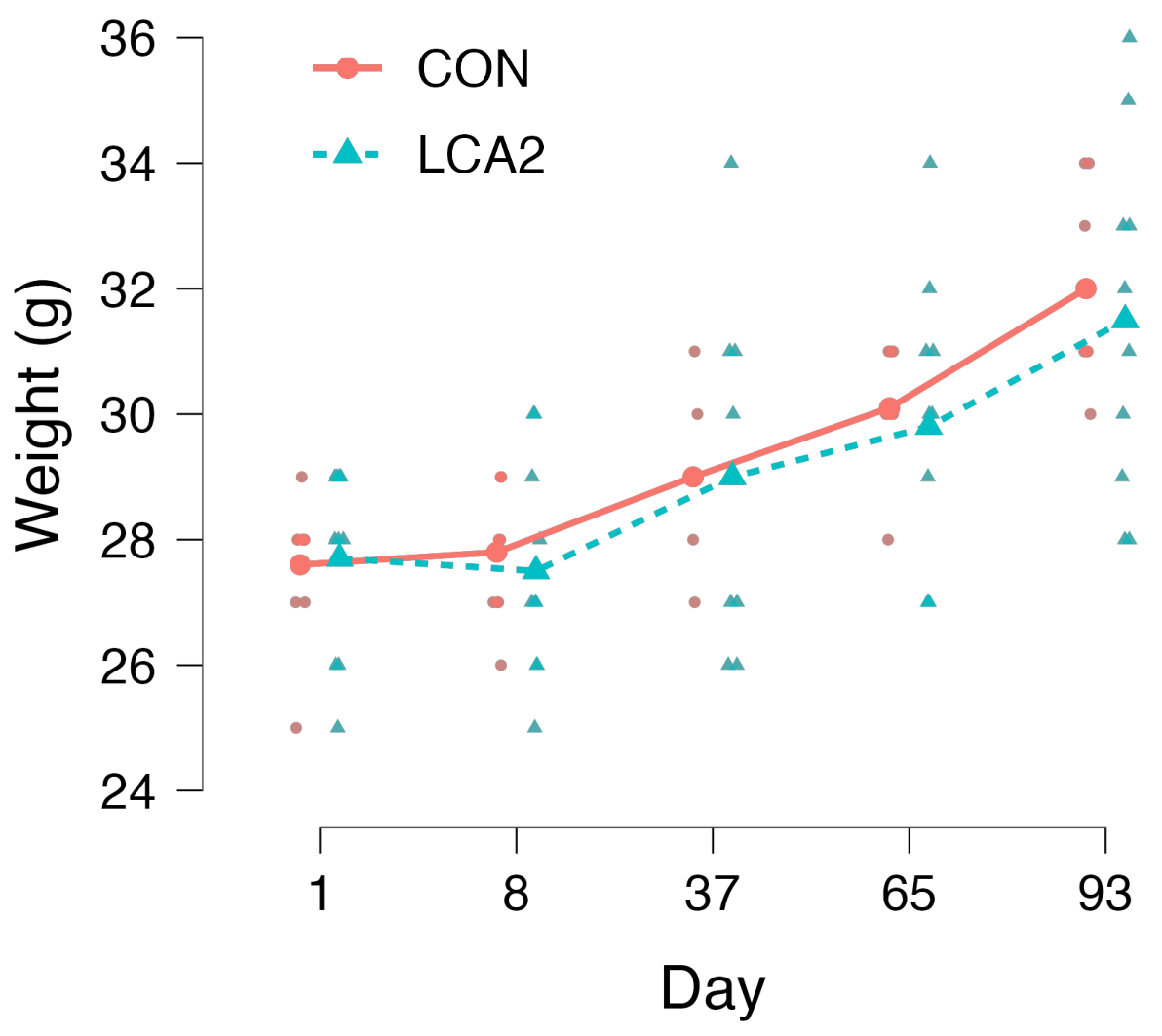

Supplement: Supplementary Figure 3 — The weight of the third cohort of mice on the days of the two initial injections and the three booster injections. [file Image_3.TIFF]

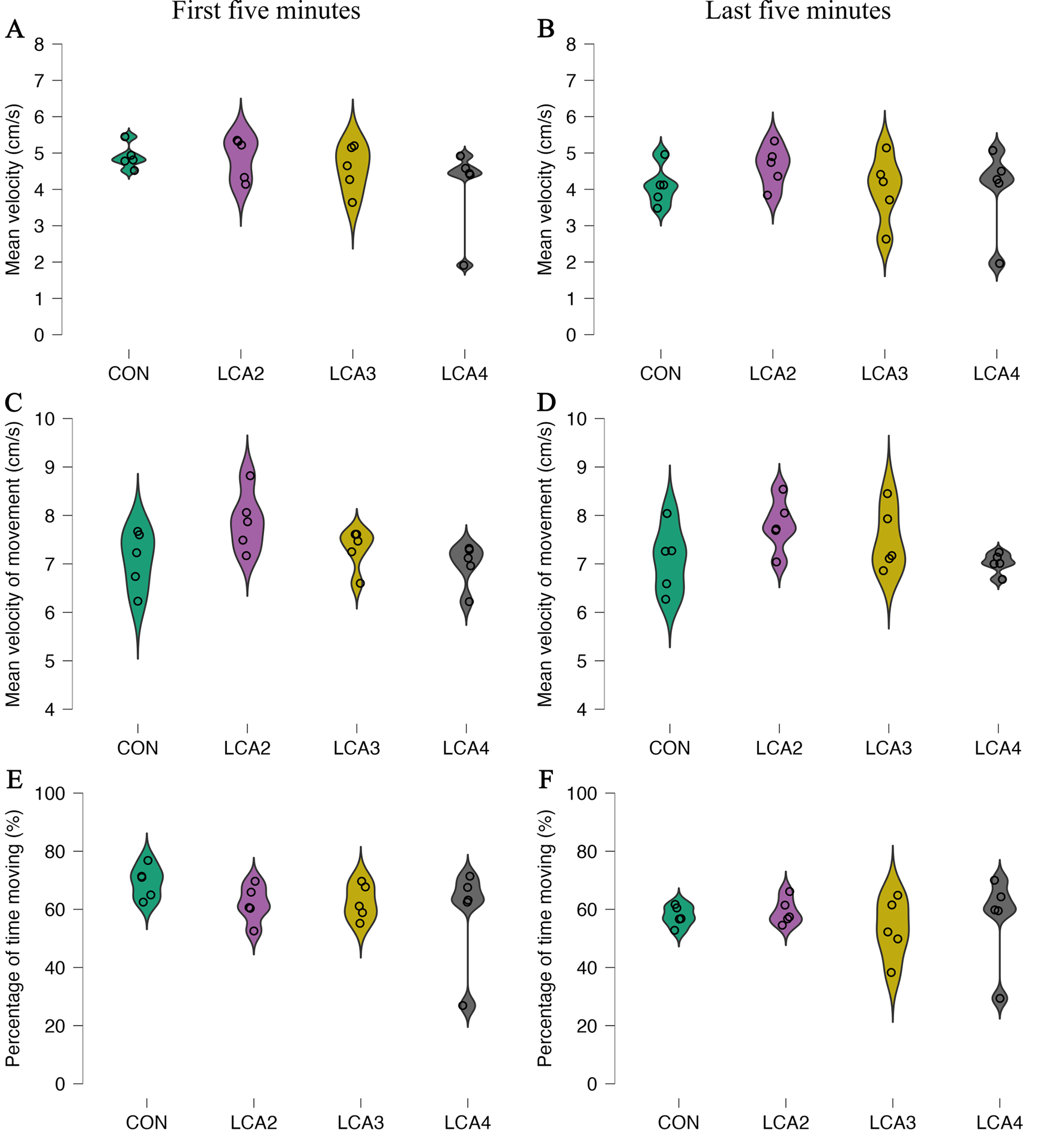

Supplement: Supplementary Figure 4 — The results of the motility parameters in the light compartment. In general, no differences were observed between the groups in any time interval for all parameters. (A,B) The distance moved relative to the time spent in the light compartment. (A) Group means: CON = 4.9 cm/s [95% CI (4.6, 5.2)], LCA2 = 4.9 cm/s [95% CI (4.4, 5.4)], LCA3 = 4.6 cm/s [95% CI (4.0, 5.2)], LCA4 = 4.1 cm/s [95% CI (3.0, 5.1)]. Welch’s ANOVA found no statistically significant difference in the light compartment during the first 5 min [F(3,8.28) = 0.85, p = 0.502, ω2 = 0.05]. The estimated effect size between CON and LCA2 was 95% CI d [−1.87, 1.94], CON and LCA3 was 95% CI d [−1.50, 2.33], CON and LCA4 was 95% CI d [−0.89, 3.01], LCA2 and LCA3 was 95% CI d [−1.54, 2.29], LCA2 and LCA4 was 95% CI d [−0.92, 3.06], and LCA3 and LCA4 was 95% CI d [−1.25, 2.63]. (B) Group means: CON = 4.0 cm/s [95% CI (3.6, 4.6)], LCA2 = 4.6 cm/s [95% CI (4.1, 5.1)], LCA3 = 4.0 cm/s [95% CI (3.2, 5.0)], LCA4 = 4.0 cm/s [95% CI (3.0, 5.0)]. No main effect was found in the light compartment [F(3, 8.59) = 0.93, p = 0.468, ω2 = 0.00]. The estimated effect sizes between CON and LCA2 was 95% CI d [−2.57, 1.30], CON and LCA3 was 95% CI d [−1.82, 1.99], CON and LCA4 was 95% CI d [−1.79, 2.02], LCA2 and LCA3 was 95% CI d [−1.22, 2.66], LCA2 and LCA4 was 95% CI d [−1.19, 2.70], and LCA3 and LCA4 was 95% CI d [−1.87, 1.93]. (C,D) The distance moved relative to time spent moving in a the light compartment. (C) Group means: CON = 7.1 cm/s [95% CI (6.6, 7.6)], LCA2 = 7.9 cm/s [95% CI (7.3, 8.4)], LCA3 = 7.3 cm/s [95% CI (6.9, 7.7)], LCA4 = 7.0 cm/s [95% CI (6.6, 7.4)]. The Welch’s ANOVA found no statistically significant main effect in the light compartment [F(3, 8.78) = 2.10, p = 0.172, ω2 = 0.21]. The effect sizes between the CON and LCA2 was 95% CI d [−3.53, 0.58], CON and LCA3 was 95% CI d [−2.32, 1.51], CON and LCA4 was 95% CI d [−1.70, 2.12], LCA2 and LCA3 was 95% CI d [−0.91, 3.06], LCA2 and LCA4 was 95% CI d [−0.42, 3 [file Image_4.TIFF]

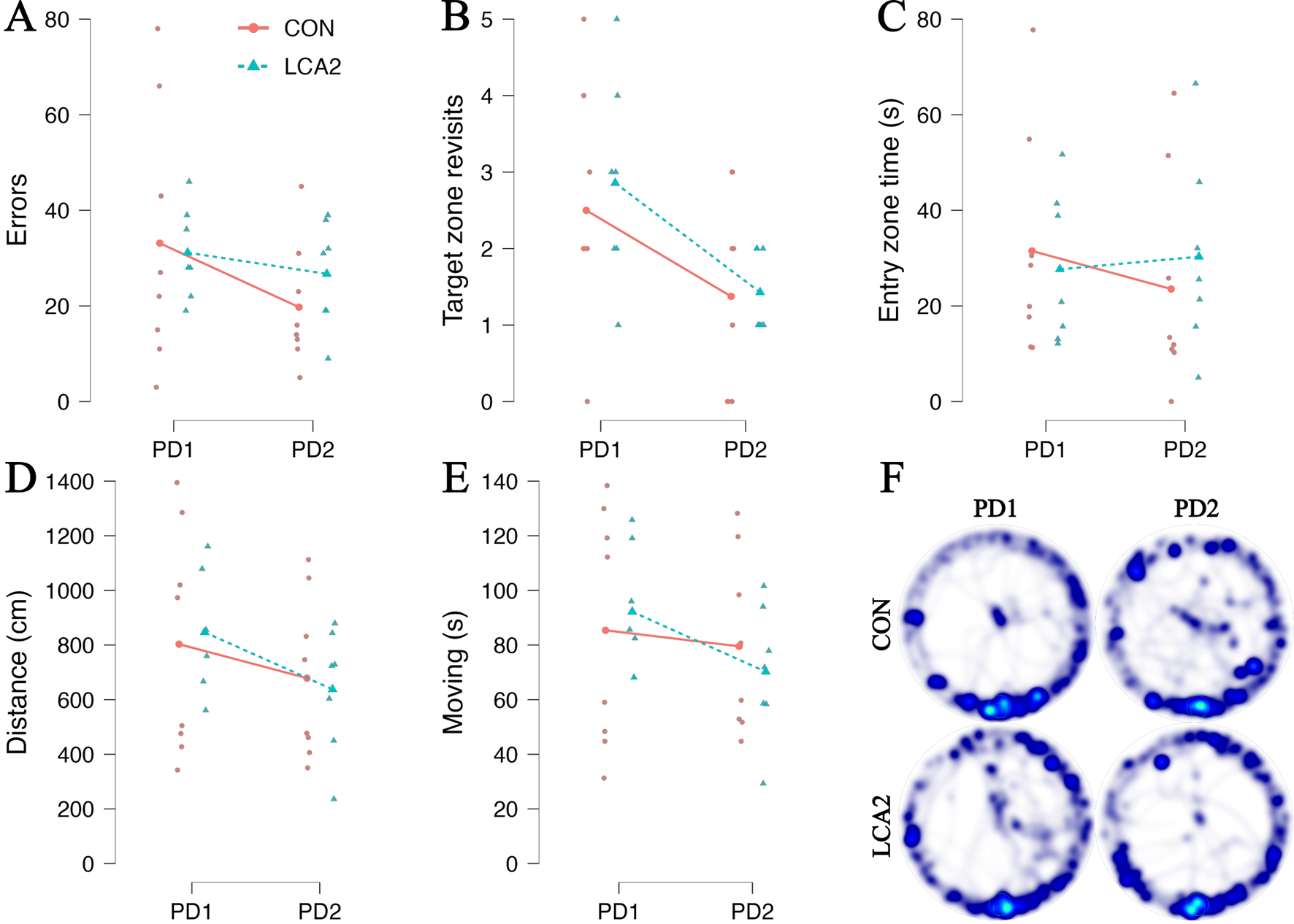

Supplement: Supplementary Figure 5 — The results from the PDs of the BM test. (A) Number of errors. (B) Number of revisits to the target zone. The escape box is removed during the PDs. Therefore, persistent visits to the zone traditionally containing the escape box reflect spatial memory. No difference was observed. (C) Time in the entry zone. (D) Distance moved. (E) Time moving. (F) Heatmaps of both groups on the two PDs. Note that the duration is fixed during the PDs as no escape box is present. Therefore, distance moved and time moving corresponds to the motility measures during TDs. [file Image_5.TIFF]

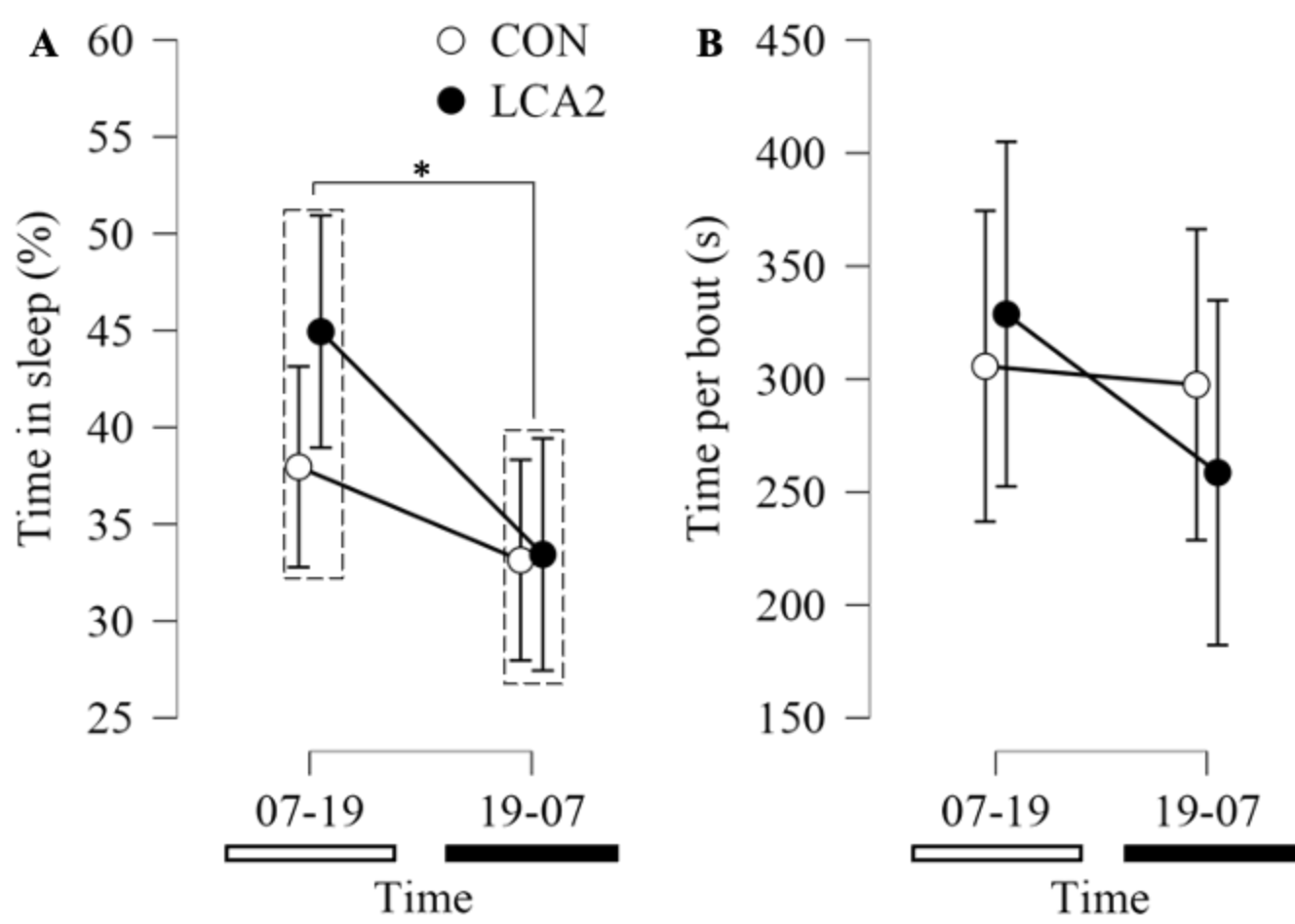

Supplement: Supplementary Figure 6 — The results from the SWM. (A) The group mean percentage time asleep during the “light on” and “lights off” periods for both the CON and LCA2 groups. A statistically significant difference was observed between time intervals. Group means: Lights on: CON = 38% [95% CI (32, 44)], LCA2 = 45% [95% CI (37, 53)], lights off: CON = 33% [95% CI (23, 43)], LCA2 = 33% [95% CI (21, 46)]. (B) The group mean duration of each sleep bout during “lights on” and “lights off” periods. Group means: Lights on: CON = 305 s [95% CI (262, 350)], LCA2 = 329 s [95% CI (245, 413)], lights off: CON = 297 s [95% CI (227, 368)], LCA2 = 259 s [95% CI (209, 308)]. The mean sleep bout duration during the 24 h of SWM was 300 s [95% CI (264, 335)] and 291 s [95% CI (249, 334)] for CON and LCA2, respectively. White bar = lights on. Black bar = lights off. Error bars = 95% CI. *p < 0.05. [file Image_6.TIFF]
